# Comprehensive analysis of multi-tissue transcriptome data and the genome-wide investigation of GRAS family in *Phyllostachys edulis*

**DOI:** 10.1038/srep27640

**Published:** 2016-06-21

**Authors:** Hansheng Zhao, Lili Dong, Huayu Sun, Lichao Li, Yongfeng Lou, Lili Wang, Zuyao Li, Zhimin Gao

**Affiliations:** 1 State Forestry Administration Key Open Laboratory on the Science and Technology of Bamboo and Rattan, International Center for Bamboo and Rattan, Beijing 100102, China; 2 Jiangxi Agricultural University, Nanchang 330045, China

## Abstract

GRAS family is one of plant specific transcription factors and plays diverse roles in the regulation of plant growth and development as well as in the plant disease resistance and abiotic stress responses. However, the investigation of GRAS family and multi-tissue gene expression profiles still remains unavailable in bamboo (*Phyllostachys edulis*). Here, we applied RNA-Seq analysis to monitor global transcriptional changes and investigate expression patterns in the five tissues of *Ph. edulis*, and analyzed a large-scale transcriptional events and patterns. Moreover, the tissue-specific genes and DEGs in different tissues were detected. For example, DEGs in panicle and leaf tissues were abundant in photosynthesis, glutathione, porphyrin and chlorophyll metabolism, whereas those in shoot and rhizome were majority in glycerophospholipid metabolism. In the portion of *Ph. edulis* GRAS (PeGRAS) analyses, we performed the analysis of phylogenetic, gene structure, conserved motifs, and analyzed the expression profiles of *PeGRAS*s in response to high light and made a co-expression analysis. Additionally, the expression profiles of *PeGRAS*s were validated using quantitative real-time PCR. Thus, *PeGRAS*s based on dynamics profiles of gene expression is helpful in uncovering the specific biological functions which might be of critical values for bioengineering to improve bamboo breeding in future.

Bamboo, as one of the large woody grasses on earth, belongs to Bambusoideae within Poaceae that includes rice, maize, wheat and other cereals^1^. As most important non-timber forest resources and fast-growth plants, a total of 75 genera bamboos including 1,250 species were identified in tropical and subtropical areas, playing a crucial role in forest ecosystems^2^. As one of the most important non-timber forest resources and forest products, moso bamboo (*Phyllostachys edulis*) is a large woody bamboo that has ecological, economic and cultural value in Asia, which accounts for ~70% of the total bamboo growth area and 5 billion US dollars of annual forest production in China^3^. Moso bamboo has broadly usages, such as timber, paper, art ware, and the shoots as delicious food. As an significant application, the timber of moso bamboo has been broadly utilized in bamboo textile industry for its physical properties^4^. It is an important for increasing farmer income and promoting local economic development in bamboo growth and production area. Moreover, with the publication of the draft genome and genomic database of moso bamboo^3^,^5^, moso bamboo has been received increasing attention in recent years.

The high-throughput RNA sequencing (RNA-Seq) represents one of essential next-generation sequencing technologies, which reveal a snapshot of RNA presence and quantity from a genome at a given moment in time^6^,^7^. As a cost-effective and a high-throughput tool, deep RNA-Seq obtains a comprehensive set of transcribed regions in the genome and yields an amount of information, such as gene expression, novel splice junctions, novel transcripts, and alternative splicing^8^. Especially, based on the achievement of the draft genome sequence of moso bamboo^3^, these sequences will facilitate to interpret functional elements and reveal molecular composition of different bamboo tissues. However, the previous studies of gene expression profiles in bamboo focused on single tissue^9^,^10^ or comparative analyses between two tissues^11^,^12^. To date, the analysis of multi-tissue transcriptome still remains elusive in bamboo.

Among the transcript factors, the GRAS gene family is important one of plant-specific. GRAS transcription factors have participated in plant growth and development processes, such as in gibberellin acid (GA) signal transduction, axillary shoot meristem formation, root radial pattering, male gametogenesis, and phytochrome A signal transduction^13^,^14^,^15^, as well as in plant disease resistance and abiotic stress responses^16^. GRAS was defined based on nuclear localization, DNA binding, and transcriptional activation features^17^,^18^. Based on considerable protein sequences, GRAS is classified into ten subfamilies and its family members have a variable N-terminal domain (N-domain) and a highly conserved C-terminal domain (GRAS-domain), which are divided into five motifs derived from the significant amino acids: leucine-rich region I (LRI), VHIID, leucine-rich region II (LRII), PFYRE and SAW^19^,^20^. Moreover, as the increasing species have their complete genome sequenced, the genome-wide analyses for GRAS family had performed in nearly 30 plant species^21^, such as Arabidopsis, rice, Chinese cabbage, pine, tomato, poplar, *Prunus mume* and so on^22^,^23^,^24^,^25^,^26^,^27^,^28^. These provide the possibility of the genome-wide comparative analysis of GRAS family members for evolutionary analysis. Despite the important roles of these genes in plant growth regulation, no findings on the GRAS genes of bamboo have been reported.

Here, we identified a large number of expressed genes in deeply sequencing pool based on RNA-Seq data from the five tissues (leaf, rhizome, root, shoot and panicle) of moso bamboo and further analyzed the bamboo GRAS family with three bamboo leaf samples under high light, both using the Illumina HiSeq 2000 sequencing platform. The tissue-specific genes and DEGs in different tissues were detected, the expression patterns of genes involved in mainly biological processes were further analyzed. Moreover, combined with RNA-Seq data, we applied a considerable analysis of bamboo GRAS members, including sequence phylogeny, gene structure, conserved motifs as well as performed the analysis of *PeGRAS* expression profiles in response to high light and a co-expression analysis. These results maybe build a foundation for dissecting the gene expression profiles in different tissues of bamboo, provide the evidence of *PeGRAS*s in response to high light, and offer a genome-wide insight into GRAS gene function, which would be helpful in selecting genes for future bamboo breeding via genetic engineering.

## Results

### Summary of the bamboo transcriptome

To effectively expand our understanding of a global gene expression profile in bamboo, high-throughput RNA-Seq using Illumina HiSeq 2000 was performed on the five tissues of moso bamboo, including leaf (LF), rhizome (RH), root (RT), shoot (SH), and panicle (PN). The statistics shown approximately 668 million reads (~65 Gb) raw data were produced, with an average of 134 million reads (~13 Gb) per tissue. Before short reads alignment, the adaptor sequences and low quality reads were trimmed during sequence preprocessing. Lastly, about 602 million reads (~59 Gb) were acquired as high quality reads. As a significant step for RNA-Seq analysis, clean reads were aligned to the reference genome from BambooGDB^5^ (Bamboo Genome Database, www.bamboogdb.org) to estimate the profile of expressed genes in each library. The widely used software of TopHat^29^ was employed for sequence alignment. In total, the output demonstrated that ~560.4 million reads, accounted for up to 93.09% of total clean reads, were mapped to the bamboo genome (Table 1). Approximately 6.91% of total reads did not align to the reference genome, likely indicating either setting strictly parameters of alignment, sequencing/assembling errors, gaps in the current genome, or alternative splicing that exists in the reference genome. Moreover, 70.20%, 38.70% and 54.38% of total reads were mapped as perfect match, unique match and multiple position matches, respectively.

### Analysis of expressed genes

RNA-Seq data could be used in the quantitative analysis of gene expression levels, determined by Fragments Per Kilobase of gene per Million mapped fragments (FPKM). To detect expressed genes in each tissue, we investigated the distribution of gene expression values among the five tissues (see Supplementary Fig. S1). The statistics of expressed genes revealed that the genes of FPKM > 0 accounted for ~91% of the total annotated genes and ~43% of expressed genes were considered as low expression values (0 < FPKM < 1). However, the number of genes with the moderate expression values (1 < FPKM ≤ 100) and high expression values (FPKM > 100) accounted for ~48% of the total annotated genes. Ultimately, the genes with FPKM ≥ 1, ranging from 14,596 (45.63%) to 16,023 (50.09%) in the five tissues, were considered as expressed genes in this study.

Relying on the comparative analysis of expression profile in five tissues, 26,328 (82.31%) and 15,488 (48.42%) genes of the total 31,987 annotated genes in bamboo genome were expressed at a statistically significant value (FPKM ≥ 1) among the different tissues (marked as among-tissue) and within an individual (marked as within-tissue), respectively. Moreover, 209 genes were not expressed (FPKM = 0) in all tissues and 27 expressed genes were detected only in one tissue (Table 2). The majority of expressed genes in the five tissues provided a significant resource for the dynamic profiles of gene expression in bamboo. Furthermore, an enrichment analysis of Gene Ontology terms was performed using all bamboo genes as the background to explore conservatively biological functions for expression genes in within-tissue (see Supplementary Table S1). Then, the results illustrated that some expressed genes were highly enriched in the processes related to “translation (GO: 0006412)”, “catabolic component (GO: 0005575)” and “structural molecule activity (GO: 0005198)”.

### Change of expressed genes in the different tissues

**Figure 1:**
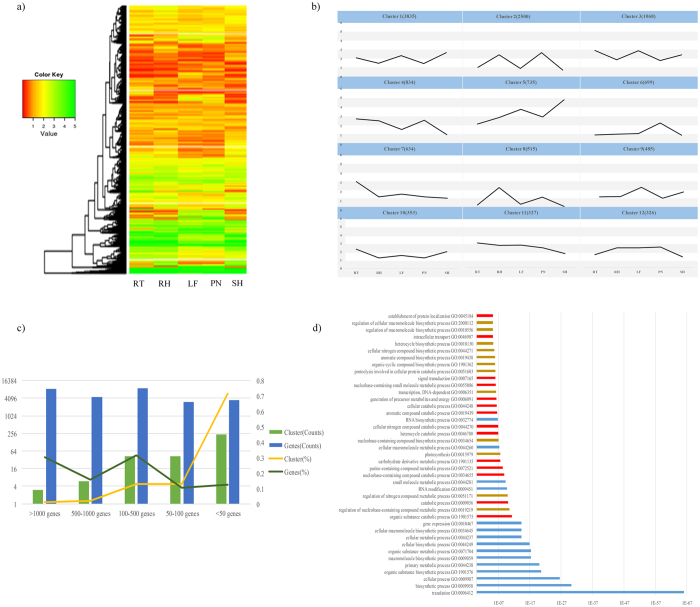
Cluster analysis of gene expression in moso bamboo. (**a**) The heat map, based on hierarchical analysis using the log_2_ (FPKM + 1) for each gene at five tissues (RT, RH, LF, PN, and SH), depicted that 21,990 expressed genes were grouped into 298 gene-expression clusters. The colors were ranging from red to bright green, representing the values of log_2_ (FPKM + 1). (**b**) The top 12 groups were identified via average value of log_2_ (FPKM + 1). The number of gene in each groups were shown in bracketed. (**c**) The overview of clustering result in different tissues, and (**d**) Significantly enriched GO categories for the top 3 clusters.

We performed the clustering affinity search technique (CAST)^30^ to elucidate dynamic transcriptome changes in the different tissues of moso bamboo. The results showed 15,488 expressed genes in within-tissue were clustered into 298 groups, with gene numbers within the clusters ranging from 1 to 3835 (Fig. 1a). As shown in Fig. 1b, the cluster less than 50 genes was dominated and accounted for 77.93% of all clusters, while only 3 clusters had more than 1000 genes, in which the number of genes accounted for more than 33% of total expressed genes. On the other hand, 14% clusters (42 clusters) had more than 100 genes. Of these, the top number of genes appeared in the cluster of 100–500 genes (Fig. 1c).

According to the cluster analysis results, some groups of expressed genes shared differentially expressed patterns. Thus, GO enrichment was analyzed in the top 3 clusters to increase our understanding of gene functions. The result indicated that some key biological processes were concentrated in the top 3 clusters, respectively (Fig. 1d and see Supplementary Table S2–4). For instance, “translation (GO: 0006412)” and “biosynthetic process (GO: 0009058)” were enriched in the Cluster 1. The enrichment of the Cluster 2 contained “regulation of nucleobase-containing compound metabolic process (GO: 0019219)” and “nitrogen compound metabolic process (GO: 0051171)”. “Organic substance catabolic process (GO: 1901575)” and “catabolic process (GO: 0009056)” were relatively abundant in the Cluster 3.

### Expression patterns in mainly biological processes

**Figure 2:**
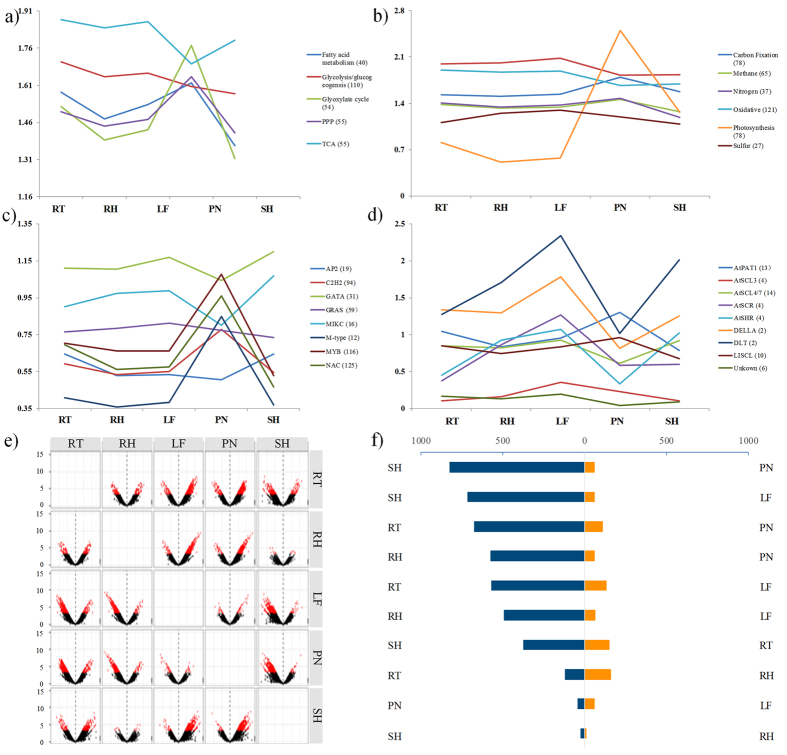
Expression patterns of 28 different families/subfamilies or groups in central metabolic pathways, energy metabolism, transcription factors, and GRAS subfamilies and the statistics of differential expression genes. The categories of pathways were based on BambooGDB. The number of gene in each families/subfamilies or groups were shown in the bracketed. X-axis was information on tissue, and Y-axis was the average value of log_2_ (FPKM + 1) in individual tissue. (**a**) The central metabolic pathways included the glyoxylate cycle, glycolysis/glucogeogensis, TCA cycle, pentose phosphate pathway (PPP) and fatty acid metabolism. (**b**) The energy metabolism included carbon fixation in carbon fixation pathways, oxidative phosphorylation, methane metabolism, nitrogen metabolism, photosynthesis and sulfur metabolism. (**c**) The transcription factors included TF families of AP2, C_2_H_2_, GATA, GRAS, MIKC, M-type, MYB and NAC, and (**d**) The GRAS family included subfamilies of AtPAT1, AtSCL3, AsSCL4/7, AtSCR, AtSHR, DELLA, DLT, LISCL, and unknown. (**e**) A volcano plot of demonstrated significant expressed genes for a pair of tissues. X-axis: log_2_ (fold change), Y-axis: −log (*p*-value), red dot: significant expression, black dot: no significant expression. (**f**) The distribution of up/down regulated genes via paired comparison.

To comprehensively unveil expression patterns of gene families or groups, we had selected and analyzed 28 different families or groups based on the pathway predicted by BambooGDB in moso bamboo. The results indicated different families or groups possessing diverse expression patterns. In Fig. 2a, the five groups could be approximately classified into two types based on similar expression patterns. One type represented TCA cycle and glycolysis cycle. Another type composed of pentose phosphate pathway (PPP), glycolysis/glucogeogensis and fatty pathway, in which the maximum and minimum expression emerged in PN and SH. Another feature was that the expression values had a rapid rise and sharply drop in LF to PN and PN to SH, respectively. In the energy metabolism (Fig. 2b), some significant changes of expression value were undetected in carbon fixation pathway, oxidative phosphorylation, methane metabolism, nitrogen metabolism and sulfur metabolism. Nevertheless, the maximum value of photosynthesis pathway appeared in PN and all the genes in other tissues were obviously down-regulated relative to the panicle sample, indicating the photosynthesis pathway played a vital role in panicles during energy metabolism.

Based on the classification of TF family, eight TF families were selected to analyze the expression patterns (Fig. 2c). The results illustrated that the four TF families, including MYB, NAC, M-type and C_2_H_2_, shared similar expression patterns, in which maximum expression values were found in PN. Whereas the expression values of other TF families in PN, containing GATA, MIKC, GRAS and AP2, were relatively lower than those of other tissues. The expression value of MIKC exhibited an obviously change in PN, indicating that MIKC may play specific roles in panicles. As the subfamilies of GRAS family (Fig. 2d), the expression value of DLT, DELLA, AtSCR and AtSHR had great variations in different tissues and shared similar expression patterns, in which maximum and minimum expression value appeared in LF and PN, respectively. However, the expression value of AtPAT1 and LISCL were higher in PN than in other tissues.

### Analyzing differentially expressed genes

Differentially expressed genes (DEGs) were identified based on pair-wise comparison between different tissues, utilizing the following cutoff: fold change ≥2 and q-value <0.05. Thus, the result showed that 3,038 genes were defined as DEGs, accounted for ~0.46% (3,038/665,297) of all pair-wise comparisons (Fig. 2e and see Supplementary Table S5). As shown in Fig. 2f, the distribution of DEGs depicted 1,607 up-regulated genes and 1,431 down-regulated genes by the paired comparison. The up-regulated genes accounted for 52.90% of all DEGs, in which the set of DEGs was majority in PN and SH. However, the down-regulated DEGs were mainly distributed in RT and PN. Moreover, a total of 173 genes (85 up-regulated genes and 88 down-regulated genes) were identified as unique DGEs in all sets of DGEs (see Supplementary Table S6).

**Figure 3:**
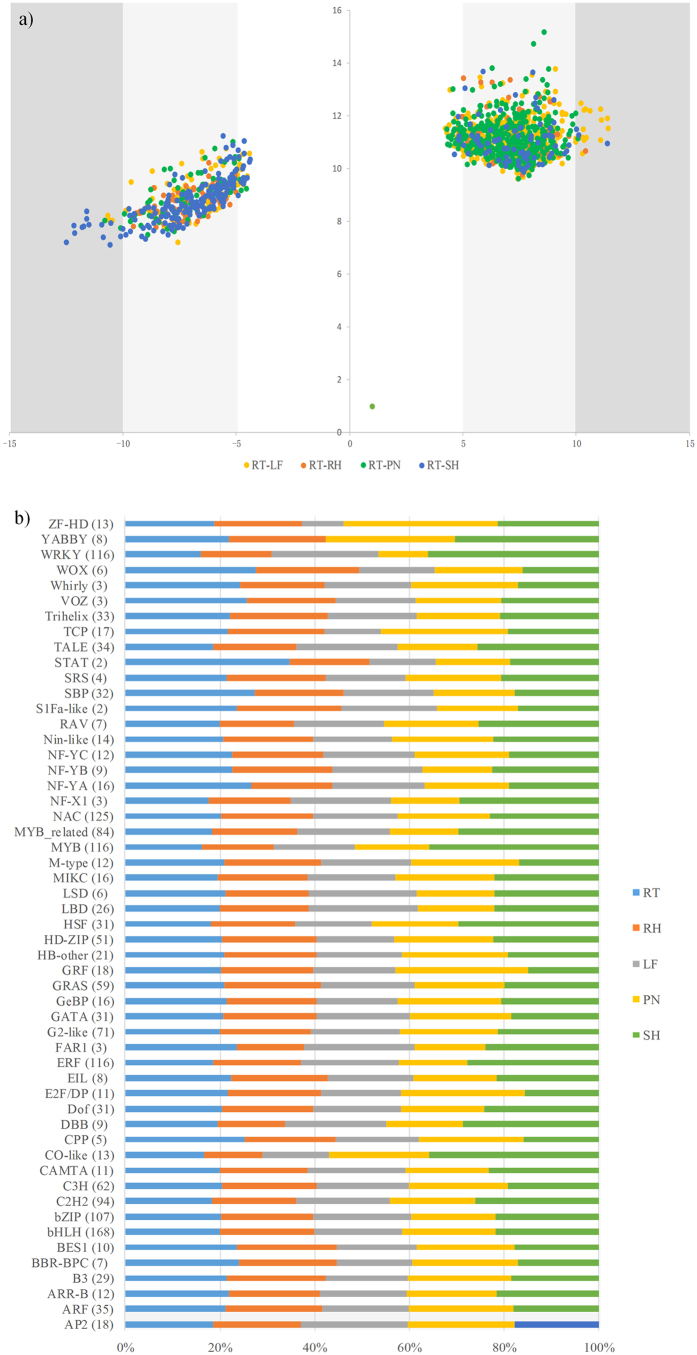
The log_2_ (fold change) distribution analysis of genes compared with root and other tissues and the average expression profiles of transcription factor families. (**a**) The X-axis are log_2_ (fold change) of the corresponding expression value compared with that of the RT, and Y-axis are log_2_ FPKM + 10 of expressed genes in LF, RH, PN, and SH. The left and right area depicted the down-regulated genes and up-regulated genes compared with RT and other tissues, respectively. The region with light gray represented the up-/down- regulated genes with 5 to10 fold-change, and the region with dark gray represented the up-/down- regulated genes more than 10 fold-change. (**b**) Due to HRT-like without expression value in five tissues, HRT-like did not show in the figure. The number of gene in each family was shown in brackets.

To deep investigate significant expression genes, we applied the log_2_ (fold change) distribution analysis by compared with RT *vs.* other tissues (Fig. 3a). The result, thus, indicated the number of up- and down-regulated DEGs was majorly distributed in 5–10 fold-changes, showing the large part of DEGs within RT *vs.* other tissues had obvious differences. After filtering duplicated genes, 942 and 517 genes were identified as the up- and down-regulated genes in RT *vs.* other tissues, respectively. Of all 78 genes involved in photosynthesis metabolism, 55 genes were detected as the up-regulated genes. Except the photosynthesis metabolism, some genes in responses to stresses were also found, such as PH01000000G3800 (heat stress transcription factor B-1), PH01000081G0140 (heat stress transcription factor), PH01000174G0590 (heat stress transcription factor), PH01000239G0570 (abscisic stress-ripening), PH01000944G0560 (stress responsive A/B Barrel domain containing protein), and PH01004112G0160 (stress responsive protein). In the down-regulated genes, 14 genes involved in phenylalanine metabolism were mainly enriched, followed by plant hormone signal transduction and flavonoid biosynthesis. For instance, the genes of PH01002904G0190 and PH01002231G0280 in phenylalanine metabolism were found in each pair-wise comparison. In total, these DGEs maybe perform crucial functions in bamboo growth and development.

### Analysis of transcription factors (TFs)

Relying on the annotation of protein function and protein structure in BambooGDB, we identified 1,768 TFs classified into 54 families in moso bamboo. The result was consistent with the prediction by PlantTFDB^31^ (see Supplementary Table S7). As shown in Fig. 3b, the average expression value of TF families in all tissues displayed various expression profiles. For example, the average expression of WRKY in shoot was much higher than that of other tissues, while one of WRKY was relatively lower expression in panicle. Moreover, the expression value of HRT-like was undetected in the five tissues and YABBY was not found in LF.

As a plant-specific transcription factor family, GRAS played critical roles in multifarious processes and was regarded as a large family in Arabidopsis, rice, and Chinese cabbage^20^,^22^,^23^. A total of 59 genes encoding GRAS proteins were identified based on BambooGDB (Table 3). In order to compare with the number of GRAS in other species, we selected 75 plants including monocots and dicots (see Supplementary Table S8), totally 3,961 GRAS proteins were analyzed, in which the largest number was in *Populus trichocarpa* (151). The number of GRAS members in moso bamboo was more than that of *Brachypodium distachyon* (48), *Triticum aestivum* (56), *A. thaliana* (37), *Cucumis sativus* (43), *Brassica rapa* (48), *Solanum lycopersicum* (54), *Prunus mume* (46) and *Vitis vinifera* (43), whereas it was less than other species, such as *Zea mays* (104), *Sorghum bicolor* (86), and *Malus domestica* (127).

### Phylogenetic analysis of GRAS

**Figure 4:**
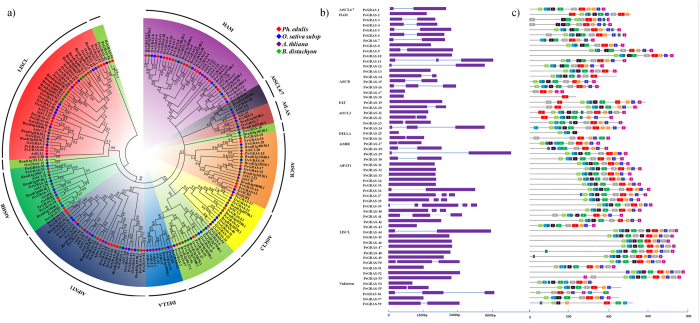
Phylogenetic analysis of GRAS and conserved motifs in GRAS proteins. (**a**) Phylogenetic tree analysis of GRAS family in *Ph. edulis*, *O. sativa* subsp*. Indica, A. thaliana*, and *B. distachyon*. The ten subfamilies included AtLAS, AtSCL4/7, HAM, AtSCR, DLT, AtSCL3, DELLA, AtPAT1, AtSHR, and LISCL. (**b**) Gene structure and motif compositions of GRAS members in *Ph. edulis* A. Exon-intron structures of GRAS genes from *Ph. edulis.* The purple boxes represent exons, and the black lines represent introns, and (**c**) Schematic representation of the conserved motifs in GRAS proteins from *Ph. edulis*. The colored boxes represent the different motifs. The non-conserved domains are represented by the black lines.

To genome-widely explore the phylogenetic relationship for GRAS TFs among the four plants, including *Ph. edulis*, *O. sativa* subsp*. Indica*, *A. thaliana*, and *B. distachyon* (Table 3), we applied Neighbor-Joining method to construct the phylogenetic tree. According to the previous report^32^, GRAS family was divided into ten subfamilies, containing AtLAS, AtSCL4/7, HAM, AtSCR, DLT, AtSCL3, DELLA, AtPAT1, AtSHR, and LISCL (Fig. 4a). However, the number of GRAS proteins in *Ph. edulis* (6), *O. sativa* (8) and *B. distachyon* (7) was unavailable in the ten subfamilies, respectively, indicating these TFs may belong to a new subfamily.

Based on the phylogenetic tree, the member of GRAS in different subfamilies has various characteristics. Among the ten subfamilies, numerous members were concentered in HAM and AtPAT1, while fewer one was AtSCL4/7 in moso bamboo. The comparative result showed that the abundant members were in HAM, AtPAT1, and LISCL subfamilies, whereas the few members in AtLAS, AtSCL4/7, and DLT subfamilies. Moreover, the GRAS subfamilies with more than 13 members were HAM (*Ph. edulis*), AtPAT1 (*Ph. edulis*), and LISCL (*B. distachyon*).

### Analysis of gene structure and conserved motifs in bamboo GRAS

Gene structural diversity is a mechanism for evolution of multi-gene families. For the further studies on the structural diversity of GRAS genes, we analyzed the coding sequences with relevant genome sequences of each GRAS gene in moso bamboo. A detailed diagram of the exon-intron structures was shown in Fig. 4b except for *PeGRAS-58*. The result demonstrated that the members in different subfamilies had different numbers of intron. For example, HAM subfamily contained one or no introns with exception of *PeGRAS-11* including two introns. Whereas, it was diverse in AtPAT1 subfamily that the largest number of intron was four. Most GRAS members had a similar exon-intron structure, indicating that these conserved intron structures may be necessary for the regulation of GRAS gene expression^33^.

GRAS proteins have the five distinct sequence motifs in the C-terminal domain, including LRI, VHIID, LRII, PFYRE and SAW. Among these, the LRI and LRII in the GRAS proteins are two leucine-rich zones. In the most cases, LRI contains a putative nuclear localization signal. A VHIID motif flanked by LRI and LRII presented in all members of GRAS proteins. The previous studies suggested that LRI, VHIID, and LRII motifs played a critical role in binding between GRAS proteins and their DNA or protein partners. A PFYRE motif consists of three units: P, FY, and RE, and the SAW motif is characterized by three pairs of conserved residues: R-E, W-G, and W-W^20^. Since amino-acid sequence patterns having various biological significances, ten GRAS protein sequences of moso bamboo were chosen to verify the five conserved motifs (see Supplementary Fig. S2).

To further analyze motif compositions of GRAS in moso bamboo, ten conserved motifs were predicted by MEME program (see Supplementary Fig. S3). Most of the GRAS proteins in the same subfamily had conservative motifs, suggesting that they own similar functions. Most of the GRAS proteins in moso bamboo had the ten conserved motifs including VHIID, PFYRE, SAW and so on. However, several members had incomplete motifs. For instance, PeGRAS-18 had only one motif. Thus, the similarity of gene structures and motifs of GRAS proteins provided evidences for the analysis of phylogenetic tree. Moreover, the diversity of motifs compositions between the subfamilies suggest that the functions of GRAS in moso bamboo are diverse.

### Expression profiles of *PeGRAS*s in response to high light

The studies shown many GRAS proteins have been participated in biotic/abiotic stresses, showing various response under different environmental conditions and treatments^16^. To deeply investigate *PeGRAS*s in response to high light and provide the valuable evidences for GRAS genes in response to abiotic stresses, we have performed high-throughput RNA-Seq for three samples of moso bamboo leaves treated with high light (1200 μmol·m^−2^·s^−1^) for 0 h (CK), 0.5 h (0.5H), and 8 h (8H). As shown in Supplementary Fig. S4(a), the cluster results of expression value shown 12 *PeGRAS*s were detected high expression (FPKM >10 in each sample) and 21 *PeGRAS*s were in lower expression value (FPKM <1 in a sample), suggesting that theses low expressed gene may also perform the essential functions. In addition, with the increased time of high light, *PeGRAS*s continued to be expressed, the down-regulated *PeGRAS*s were abundant in 0.5H and the up-regulated ones were concentrated in CK and 8H, indicating that the expression of *PeGRAS* may be first repressed in short-term high light, then the expression reach the normal value. Moreover, the results of CAST clustering in Supplementary Fig. S4(b) indicated all *PeGRAS*s were grouped into 6 groups based on expression pattern. Though identifying different expressed patterns for *PeGRAS*s in response to high light, many *PeGRAS*s (~63%) shared one pattern (Cluster 5). These evidences may indicate that *PeGRAS*s exhibit various modules in response to high light, but mainly response pattern was one module, *i.e.* expression value first declined in initial stage of high light, then ascended during the increasing time of high light. The result of CAST clustering was consistent with that of expression value. Therefore, these findings will help us expand the knowledge for GRAS genes in response to high light stress.

We had performed a co-expression analysis using the relative values of expression in above RNA-Seq data, centered on the average expression, based on a Pearson Correlation Coefficient (PPC) with the cutoff of 0.2^25^,^34^. The clustered genes were considered under same pattern possessing similar regulatory elements. Thus, the results shown that 56 genes, including 3 *PeGRAS*s, were in the co-expressed gene set, which involved in some TFs, plant hormone signal transduction, ribosome metabolism, and transporters. The annotations of the co-expressed gene set were provided in Supplementary Table S9.

## Discussion

Due to the complexity of reference genome as well as limitations of sequencing and alignment methods, it was unavoidable that some reads aligned multi-position. These were ultimately mapped into one position of reference genome randomly, causing some biases in the evaluation of gene expression levels. To obtain better alignment results, the parameters of TopHat were properly set based on genomic architecture and library construction, such as inner distance and standard deviation. In this study, we calculated the two parameters via Trinity and Picard software in each library (see method) (Table 1). Through a comparative analysis of TopHat with/without the two parameters (see Supplementary Table S10 and Fig. S5), the alignment result showed the total number of unique match was increased by 37,787,739 reads (~6.27% of total reads), whereas the average multi-position match was reduced by 19,240,798 reads (~3.19% of total reads). Multi-position matches in the read mapping maybe connect to ancient whole genome duplication. Moreover, a total of the mapping reads was increased by ~3.08%, the unmapped reads was reduced by ~3.08%. In sum, the alignment results were improved by accurately set the two parameters, *i.e.* inner distance and standard deviation. It contributed to yield more reliable data for further analyses such as expression profile and alternative splicing.

The previous study considered genes exhibiting stable expression profiles in tissues as potential housekeeping genes^35^. Thus, based on the hypothesis, we identified 422 genes with co-variance values less than 0.3 as potential housekeeping genes in different tissues (see Supplementary Table S11) combined with available RNA-Seq data of moso bamboo^11^,^12^,^36^. Moreover, we also defined 270 genes of *Arabidopsis thaliana*^37^ and 415 genes of *Oryza sativa*^38^ as the reciprocal best genes with candidate housekeeping genes in moso bamboo, respectively. The results illustrated the pathways contained the candidate housekeeping genes were mainly concentrated in the central pathways (carbohydrate metabolism, energy metabolism, lipid metabolism), transport, translation, and signal metabolism. Then, the dataset of the potential housekeeping genes may be valuable for experimental studies on gene function and regulation in future.

Tissue specific genes in different tissues indicated they may all be functionally related to the tissue growth, development, and metabolic processes. A total of 27 tissue specific genes were identified, including 4 in shoot, 6 in root, 5 in panicle and 12 in leaf. The leaf specific genes were detected from several gene families, including carbonic anhydrase, potassium transporter, MATE efflux family protein and RING-H2 finger protein. These genes might be involved in carbon assimilation, ion transport and stress response. However, no rhizome specific genes were found in this study, which was different from other plants with rhizome such as *Atractylodes lancea*^39^, *Oryza longistaminata*^40^ and *Zingiber officinale*^41^. The rhizome specific genes were expected to play important roles in rhizome growth and development, thus the tissue-specific studies need to further investigate.

In the DEGs, the up-regulated genes of PN and LF were mainly concentrated in photosynthesis, glutathione, porphyrin and chlorophyll metabolism. PH01002424G0210 was identified as a DEG in PN *vs.* SH. Based on the annotation and experimental evidence from a reciprocal best gene (AT2G38310) in *A. thaliana*, PH01002424G0210 may be considered as abscisic acid sensors, classified in the pathway of plant hormone signal transduction and mainly involved in the development of panicle. Whereas the DGEs in SH and RH were found in glycerophospholipid metabolism, which maybe relate to fast-growth by accelerating cell division and cell growth in the development of shoot and rhizome. PH01002370G0230, whose annotation was transferring acyl groups other than amino-acyl groups, was defined as a unique DEG appeared in SH *vs.* RT. As a reciprocal best gene of PH01002370G0230, the gene of AT2G26640 in *A. thaliana* was annotated as function associated with the responses to cold, light and osmotic stress located in membrane indicating PH01002370G0230 located in membrane maybe play an essential role of nutrients transferring and responding to cold and light stresses in the development stage of shoot. Integrated with the growth and development of different bamboo tissues and the DEGs, we predicted that the DEGs might be involved in the regulation of material transport and metabolism in different tissues of bamboo.

Moreover, we had identified a TF network of moso bamboo based on a high-confidence *Arabidopsis* transcriptional regulatory data^42^. As shown in Supplementary Table S12 and Fig. S6, the results indicated that a total of 97 TFs from 21 families were identified in moso bamboo TFs network. The 6 interactions were found between MYB and NAC, suggesting that both TF families have close connection in transcriptional regulation. Moreover, more self-regulated interactions were detected in NAC and WRKY, suggesting that the TFs in these families may have diverse functions.

In addition, the result demonstrated that the homologous genes encoding AtLAS were not found in moso bamboo by analyzing phylogenetic tree and aligning genomic sequence. However, GRAS members in other three species were detected and clustered into the AtLAS subfamily, which inferred that moso bamboo also has the member of AtLAS subfamily. To further seek the homologous genes, we applied *de novo* transcriptomic assembling to construct the transcriptomes using Trinity (see Supplementary Table S13). The result showed the homology sequences of AtLAS were still unavailable in transcriptional level. Therefore, we intended to draw the conclusion that the homologous genes of AtLAS maybe lost in moso bamboo.

**Figure 5:**
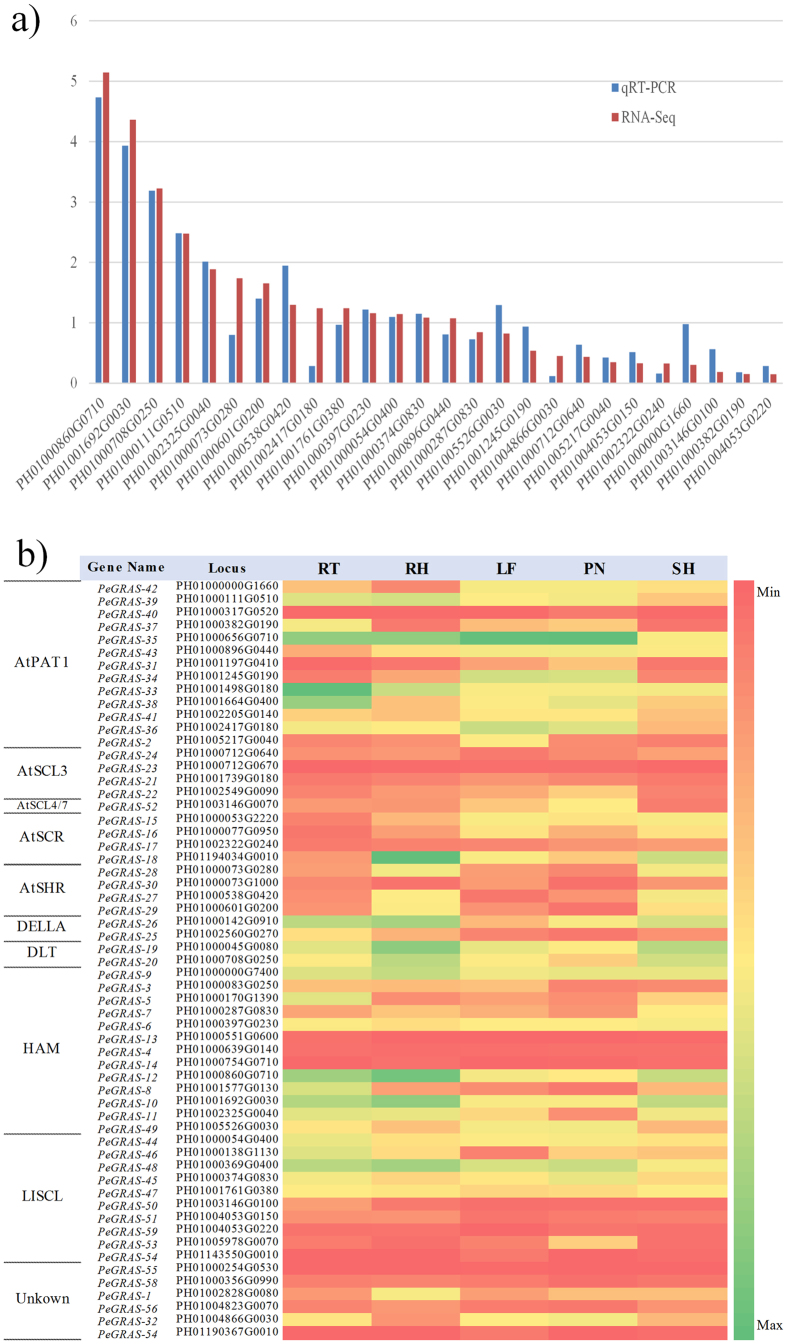
Validation of RNA-Seq data by quantitative real time RT-PCR and expression analysis of GRAS genes in different tissues of moso bamboo. (**a**) A histogram of gene expression combined RNA-Seq data and quantitative real time RT-PCR. X-axis represented 26 selected genes randomly. Y-axis represented expression value, and (**b**) A heatmap of GRAS genes expression in five tissues.

To experimentally verify the expressed genes based on RNA-Seq, quantitative real time PCR (qRT-PCR) assay was performed using independently collected rhizome tissue, which was in the same developmental stage as those used for the RNA-Seq analysis. Among the 26 selected genes randomly, the Pearson Correlation Coefficient (PCC) based on expression values between qRT-PCR and RNA-Seq data was 0.9491, showing that the RNA-Seq data were highly reliable (Fig. 5a).

In addition, we used RNA-Seq data to analyze different expression levels of GRAS genes in the five tissues of moso bamboo, indicating that their expression value statistically enhanced or repressed in the different tissues (Fig. 5b). Among the 59 genes, most of them were expressed in the different tissues. Some *PeGRAS*s were higher expression in some tissues. For instance, *PeGRAS-13* had a high transcript level in RT while *PeGRAS-2* and *PeGRAS-14* were mostly expressed in SH and RT. All of them were clustered into the HAM subfamily. It was reported that the subfamily was associated with the regulation of the shoot development in *A. thaliana*, *Petunia hybrid* and pepper^43^,^44^. Several *PeGRAS*s show the feature of tissue-specific expressions. For instance, *PeGRAS-54* was only expressed highly in LF and SH, while *PeGRAS-13* was detected in PN, RT, and SH. The genes from *PeGRAS-44* to *PeGRAS-53*, belonging to LISCL subfamily, were mostly detected in PN. LISCL regulates meiosis-associated genes in *Liliuum longiflorum*^45^. These indicated that the GRAS genes maybe involve in different mechanisms during plant growth and development.

Taken together, we applied the high-throughput RNA sequencing to investigate the global transcriptomic dynamics and DEGs in five *Ph. edulis* tissues. Moreover, a total of 1,768 TFs were identified and classified into 54 families in moso bamboo, among which 59 GRAS members were further analyzed, such as sequence phylogeny, gene structure, conserved motifs and gene expression profiles validated by qRT-PCR. We also analyzed the expression profiles of *PeGRAS*s in response to high light and a co-expression analysis. Therefore, these results may provide a key basis for further experimental research on the function of genes and TFs in regulating the growth and development of bamboo, and may help to select the genetic factors valuable for future bamboo breeding by engineering modification.

## Methods

### The material collection of moso bamboo tissues

The five tissues in moso bamboo were collected, containing leaf (LF), rhizome (RH), root (RT), shoot (SH), and panicle (PN), and 3 individual samples were gathered. Of these, four vegetative tissues (LF, RH, RT, and SH) were picked in spring from the city of Lin’an, Zhejiang Province of China (119°26′55.0′′E, 30°19′13.4′′N, 480 meter in elevation) and one reproductive tissue (PN) was gathered in autumn from the city of Guilin, Guangxi Province of China (110°31′20.2′′E, 25°10′42.7′′N, 216 meter in elevation). Moreover, to provide and expand the evidences of the GRAS genes in response to abiotic stress, we have collected three samples of moso bamboo seedlings under same high light intensities (1200 μmol·m^−2^·s^−1^) for different treated times, including 0.5 hour (0.5H) and 8 hours (8H), and 0 hour as control (CK). The moso bamboo seedlings were potted in our laboratory under long-day conditions (16 h light/8 h dark) at 25 °C, with a light intensity of 200 μmol·m^−2^·s^−1^ before high light treatment. The high light was provided by cool white fluorescent tubes. The third leaf on the top of seedlings were selected for the RNA sequencing. The detailed information was provided^46^. All samples were collected and quickly frozen in liquid nitrogen. Furthermore, it took two years to look for the floral tissues in main bamboo growth regions of China due to infrequent and unpredictable flowering events of bamboo. It is hard to collect three individual samples of panicle during the same stage in the given time. Therefore, the above situation brought about a consequence that replicated samples for panicle were unavailable by now.

### RNA isolation, cDNA library construction, and sequencing

The total RNA was isolated from five tissues using TRIZOL Reagent Solution (Invitrogen, Carlsbad, CA, USA) on the basis of the manufacturer’s instructions. The purity and concentration were detected using a NanoDrop 2000 spectrophotometer. Reverse transcription was conducted with Reverse Transcription System (Promage, USA)^47^. The extracted RNA was treated with RNase-free DNase I for 30 min at 37 °C to remove the residual DNA. cDNA library construction and normalization were performed as previously described^48^. Then, the five pooled libraries from five tissues were 2 × 100 bp by Illumina HiSeq^TM^ 2000 platform (Illumina, San Diego, CA, USA).

### *De novo* assembly of the clean reads, mapping reads to the reference bamboo genome, and estimation of gene expression

Firstly, adaptor sequences and low quality sequences were trimmed using Trimmomatic^48^ in the preprocessing. Secondly, to accurately calculate two parameters of TopHat, expected inner distance and standard deviation, we performed *de novo* assembling of the clean reads by Trinity software^49^. Then, as the reference, genome sequences and annotation of moso bamboo was downloaded from BambooGDB (www.bamboogdb.org)^5^. The filtered sequences were mapped to the reference of bamboo genome using TopHat^29^. Based on insert size calculated by Trinity and the feature of genome, some parameters were optimized. For example, max-intron-length was 2000, min-intron-length was 30, expected inner distance was listed in Table 1. The remaining parameters were utilized as default. Subsequently, the aligned read files were processed by Cufflinks^50^. After reads were assembled into transcripts, their abundance was estimated and normalized using the numbers of reads per kilobase of exon sequence in a gene per million mapped reads^51^. Owing to without replicated samples, we had applied cuffdiff program (one of programs in cufflinks) via setting the parameter of dispersion-method as ‘blind’ to detect differentially expressed genes. This configuration was suitable for a single biological replicate and was utilized in the previous studies^52^,^53^. Moreover, in the analysis of functional and structural annotation, GO enrichment was carried out using Ontologizer^54^.

### Accession numbers

All sequence data for five tissues (LF, RH, RT, SH, and PN) and three samples under high light from this article have been deposited in the Short Read Archive (SRA) at the NCBI database under the following accession numbers: SRX082501, SRX082502, SRX082503, SRX082504, SRX082507, SRX082508, SRX082509, SRX082510, SRX082511, SRR2035212, SRR2035263, and SRR2035327.

### Analysis of GRAS gene structure and construction of phylogenetic tree

The CDS and genome sequences of moso bamboo were downloaded from BambooGDB (http://www.bamboobdb.org). In addition, the exon-intron structures of genes were performed with GSDS (http://gsds.cbi.pku.edu.cn)^55^. The information of GRAS proteins of Arabidopsis and rice were referred to the previous study^32^. The GRAS protein sequences in Arabidopsis, rice and *B. distachyon* were obtained from The Arabidopsis Information Resource database (http://www.arabidopsis.org), Rice Genome Annotation Protect (http://rice.plantbiology.msu.edu) and PlantTFDB (http://planttfdb.cbi.pku.edu.cn), respectively. Moreover, all GRAS protein sequences were aligned with ClustalW. The phylogenetic tree was constructed by Neighbor-Joining method of MEGA 6 (http://www.megasoftware.net)^56^. The MEME program (version 4.9.1) was used to predict the motifs in 59 GRAS proteins in moso bamboo (http://meme.nbcr.net)^57^.

### Expression patterns of GRAS transcription factor in moso bamboo

The primers of GRAS genes from moso bamboo were designed using Primer Premier 5.0 (see Supplementary Table S14). All primers were tested with rTaq (TaKaRa, Japan). The qRT-PCR reactions were performed with a qTOWER2.2 Real-Time PCR System (Analytik Jena, Germany) using Roche LightCycler 480 SYBR Green I Master. The qRT-PCR procedure consisted of 95 °C for 10 min and 50 cycles of 95 °C for 20 s, 60 °C for 10 s. The reaction volume was 10 μL and contained 5.0 μL 2 × SYBR Green I Master Mix, 0.8 μL cDNA, 0.2 μL forward primer and reverse primer each (5 μM), and 3.8 μL ddH_2_O. All reactions were repeated three times. For each condition, the qRT-PCR experiments were performed as biological triplicates. The relative gene expression level was calculated with the 2^−△△Ct^ method^58^ using *NTB* as reference gene^59^.

## Additional Information

**How to cite this article**: Zhao, H. *et al.* Comprehensive analysis of multi-tissue transcriptome data and the genome-wide investigation of GRAS family in *Phyllostachys edulis. Sci. Rep.* **6**, 27640; doi: 10.1038/srep27640 (2016).

## Supplementary Material

Supplementary Information

Supplementary Dataset 1

## Figures and Tables

**Table 1:** Summary of RNA-Seq data based on the different tissues collected from moso bamboo.

Tissue	Expected InnerDistance (bp)^a^	StandardDeviation (bp)^b^	Total BasePairs (bp)^c^	TotalReads^d^	Total MappedReads^e^	PerfectMatch^f^	Mismatch(<=2 bp)^g^	UniqueMatch^h^	Multi-positionMatch^i^	UnmappedReads^j^
LF	229	43.29	7,940,029,694	75,061,531(100.00%)	65,799,473(87.66%)	47,086,647(62.73%)	14,953,616(19.92%)	28,517,274(37.99%)	37,282,199(49.67%)	9,262,061(12.34%)
RH	222	47.92	8,100,321,514	84,229,772(100.00%)	80,181,256(95.19%)	56,701,555(67.32%)	21,085,116(25.03%)	33,042,653(39.23%)	47,138,603(55.96%)	4,048,516(4.81%)
RT	201	54.35	7,709,128,042	73,771,970(100.00%)	65,449,065(88.72%)	45,734,234(61.99%)	16,281,541(22.07%)	24,121,167(22.07%)	41,327,898(56.02%)	8,322,905(11.28%)
SH	248	51.40	27,945,422,424	285,876,190(100.00%)	271,558,354(94.99%)	224,043,003(78.37%)	42,224,857(14.77%)	118,238,306(41.36%)	153,320,048(53.63%)	14,317,836(5.01%)
PN	216	55.51	8,065,743,156	83,872,774(100.00%)	78,158,914(93.19%)	49,616,883(59.16%)	25,431,109(30.32%)	29,373,018(35.02%)	48,785,896(58.17%)	5,713,860(6.81%)
Total			59,760,644,830	602,812,237(100%)	561,147,062(93.09%)	423,182,322(70.20%)	119,976,239(19.90%)	233,292,418(38.70%)	327,854,644(54.39%)	41,665,178(6.91%)

^a^Expected inner distance: the expected inner distance between mate pairs.

^b^Standard deviation: the standard deviation for the distribution on inner distances between mate pairs.

^c^Total Base Pairs: the number of base pairs generated from sequencing after filtering low quality reads.

^d^Total Reads: the number of reads generated from sequencing after filtering low quality reads.

^e^Total Mapped Reads: the number of reads mapped to the reference genome within 2 bp mismatch.

^f^Perfect Match: the number of reads mapped to the reference genome with no mismatch.

^g^Mismatch (<=2 bp): the number of reads mapped to the reference genome with no more than 2 bp mismatch.

^h^Unique Match: the number of reads mapped to the reference genome with unique position.

^i^Multi-position Match: the number of reads mapped to the reference genome with multi-position.

^j^Unmapped Reads: the number of reads that could not be mapped to the reference genome within 2 bp mismatch.

**Table 2:** Expressed genes detected only in the single tissue.

Locus name	Tissue		Annotation^b^	Reciprocal best gene with*O*.*sativa*^b^
	Name	FPKM^a^		
PH01000055G1420	RT	51.7245	Expressed protein	N/A^c^
PH01000061G0830	LF	1.40606	Hypothetical protein	LOC_Os06g43720.1
PH01000083G0050	RT	2.12534	Methyltransferase	N/A
PH01000462G0820	PN	2.43972	Decarboxylase	LOC_Os08g04560.1
PH01000543G0780	LF	3.47426	OsSub24 - Putative Subtilisin homologue	LOC_Os02g53840.1
PH01000722G0580	RT	1.96096	Xyloglucan fucosyltransferase	LOC_Os06g10910.1
PH01000940G0480	SH	2.42569	Transcription factor	N/A
PH01001339G0510	RT	4.92908	Hypothetical protein	LOC_Os01g05850.1
PH01001367G0280	LF	1.07199	Transporter family protein	N/A
PH01001742G0230	SH	1.35667	CSLF6 - cellulose synthase-like family F	N/A
PH01001945G0230	LF	1.71132	RING-H2 finger protein	N/A
PH01002005G0310	LF	1.23774	Potassium transporter	LOC_Os03g37930.1
PH01002440G0410	PN	1.1751	MYB family transcription factor	LOC_Os01g06320.1
PH01003129G0090	PN	1.09368	NDP1	N/A
PH01003527G0170	PN	1.92066	Cytochrome P450	LOC_Os04g08828.1
PH01003538G0010	RT	2.54772	Expansion precursor	LOC_Os05g19600.1
PH01005045G0050	LF	1.70194	Disease resistance RPP8-like protein 3	N/A
PH01006473G0020	LF	2.91698	Dirigent	LOC_Os11g10800.1
PH01013368G0010	LF	6.74763	Carbonic anhydrase, chloroplast precursor	N/A
PH01040787G0010	LF	2.61237	Expressed protein	LOC_Os03g44880.1
PH01161271G0010	SH	231.32	Hydrolase	N/A
PH01173456G0010	SH	548.909	Exosome complex exonuclease	LOC_Os09g07820.2
PH01183803G0010	PN	3.27749	9-cis-epoxycarotenoid dioxygenase 1, chloroplast precursor	N/A
PH01192415G0010	LF	4.03645	MATE efflux family protein	N/A
PH01217799G0010	LF	8.25326	Vignain precursor	LOC_Os04g13140.1
PH01268895G0010	LF	316.557	Diacylglycerol kinase	LOC_Os05g19670.1
PH01269246G0010	RT	1276.72	Helix-loop-helix DNA-binding domain containing protein	LOC_Os01g02110.1

^a^FPKM represents Fragments Per Kilobase of gene per Million mapped fragments.

^b^The annotation of bamboo and reciprocal best genes with *O. sativa* were from BambooGDB.

^c^N/A represents no available.

**Table 3:** The information of GRAS transcription factors.

Subfamily	Species^a^	Transcription factor name (Locus name)^b^
AtSCL4/7	*Ph. edulis* (1)^c^	PeGRAS-1 (PH01002828G0080)
	*O. sativa* (1)	OsGRAS-18 (Os03g51330)
	*B. distachyon* (1)	(Bradi1g10330.1)
	*A. thaliana* (2)	AtSCL4 (At5g66770); AtSCL7 (At3g50650)
HAM	*Ph. edulis* (13)	PeGRAS-2 (PH01005217G0040); PeGRAS-3 (PH01000083G0250); PeGRAS-4 (PH01000639G0140); PeGRAS-5 (PH01000170G1390); PeGRAS-6 (PH01000397G0230); PeGRAS-7 (PH01000287G0830); PeGRAS-8 (PH01001577G0130); PeGRAS-9 (PH01000000G7400); PeGRAS-10 (PH01001692G0030); PeGRAS-11 (PH01002325G0040); PeGRAS-12 (PH01000860G0710); PeGRAS-13 (PH01000551G0600); PeGRAS-14 (PH01000754G0710)
	*O. sativa* (8)	OsGRAS-20 (Os04g46860); OsGRAS-8 (Os02g44360); OsGRAS-9 (Os02g44370); OsGRAS-28 (Os06g01620); OsGRAS-52 (Os12g06540); OsGRAS-41 (Os11g06180); OsGRAS-12 (Os03g15680); OsGRAS-37 (Os10g40390)
	*B.distachyon* (7)	(Bradi1g67340.1); (Bradi4g41880.1); (Bradi4g24867.1); (Bradi1g52240.1); (Bradi3g32890.1); (Bradi1g78230.1); (Bradi3g50930.1)
	*A. thaliana* (5)	AtSCL15 (At4g36710); AtSCL22 (At3G60630); AtSCL27 (At2g44370); AtSCL6 (A4g00150); AtSCL26 (At4g08250)
AtSCR	*Ph. edulis* (4)	PeGRAS-15 (PH01000053G2220); PeGRAS-16 (PH01000077G0950); PeGRAS-17 (PH01002322G0240); PeGRAS-18 (PH01194034G0010)
	*O. sativa* (4)	OsSCR2 (Os12g02870); OsSCR1 (Os11g03110); OsGRAS-32 (Os07g38030); OsGRAS-25 (Os05g40710)
	*B. distachyon* (4)	(Bradi2g22010.1); (Bradi2g13560.1); (Bradi1g24310.1); (Bradi4g44090.1)
	*A. thaliana* (2)	AtSCR (At3g54220); AtSCL23 (At5g41920)
DLT	*Ph. edulis* (2)	PeGRAS-19 (PH01000045G0080); PeGRAS-20 (PH01000708G0250)
	*O. sativa* (1)	OsGRAS-29 (Os06g03710)
	*B. distachyon* (1)	(Bradi1g49630.1)
	*A. thaliana* (1)	AtSCL28 (At1g63100)
AtSCL3	*Ph. edulis* (4)	PeGRAS-21 (PH01001739G0180); PeGRAS-22 (PH01002549G0090); PeGRAS-23 (PH01000712G0670); PeGRAS-24 (PH01000712G0640)
	*O. sativa* (5)	OsGRAS-40 (Os11g04590); OsGRAS-51 (Os12g04380); OsGRAS-23 (Os05g31380); OsGRAS-24 (Os05g31420); OsGRAS-6 (Os01g71970)
	*B. distachyon* (3)	(Bradi2g60750.1); (Bradi4g43200.1); (Bradi4g26520.1)
	*A. thaliana* (1)	AtSCL3 (At1g50420)
DELLA	*Ph. edulis* (2)	PeGRAS-25 (PH01002560G0270); PeGRAS-26 (PH01000142G0910)
	*O. sativa* (1)	OsSLR1 (Os03g49990)
	*B. distachyon* (1)	(Bradi1g11090.1)
	*A. thaliana* (5)	AtRGL1 (At1g66350); AtRGA (At2g01570); AtGAI (At1g14920); AtRGL2 (At3g03450); AtRGL3 (At5g17490)
AtSHR	*Ph. edulis* (4)	PeGRAS-27 (PH01000538G0420); PeGRAS-28 (PH01000073G0280); PeGRAS-29 (PH01000601G0200); PeGRAS-30 (PH01000073G1000)
	*O. sativa* (5)	OsSHR2 (Os03g31880); OsSHR1 (Os07g39820); OsGRAS-13 (Os03g29480); OsGRAS-35 (Os07g40020); OsGRAS-26 (Os05g42130)
	*B. distachyon* (4)	(Bradi1g23060.1); (Bradi1g60140.1); (Bradi2g20760.1); (Bradi1g22907.1)
	*A. thaliana* (3)	AtSHR (At4g37650); AtSCL29 (At3g13840); AtSCL32 (At3g49950)
AtPAT1	*Ph. edulis* (13)	PeGRAS-31 (PH01001197G0410); PeGRAS-32 (PH01004866G0030); PeGRAS-33 (PH01001498G0180); PeGRAS-34 (PH01001245G0190); PeGRAS-35 (PH01000656G0710); PeGRAS-36 (PH01002417G0180); PeGRAS-37 (PH01000382G0190); PeGRAS-38 (PH01001664G0400); PeGRAS-39 (PH01000111G0510); PeGRAS-40 (PH01000317G0520); PeGRAS-41 (PH01002205G0140); PeGRAS-42 (PH01000000G1660); PeGRAS-43 (PH01000896G0440)
	*O. sativa* (7)	OsGRAS-1 (Os03g09280); OsGRAS-36 (Os10g22430); OsGIGR2 (Os07g39470); OsGIGR1 (Os07g36170); OsGRAS-3 (Os01g65900); OsGRAS-21 (Os04g49110); OsGRAS-10 (Os02g45760)
	*B. distachyon* (5)	(Bradi3g24210.1); (Bradi1g23350.1); (Bradi1g25370.1); (Bradi2g56910.1); (Bradi5g19190.1)
	*A. thaliana* (6)	AtSCL21 (At2g04890); AtPAT1 (At5g48150); AtSCL5 (At1g50600); AtSCL13 (At4g17230); AtSCL1 (At1g21450); AtSCL8 (At5g52510)
LISCL	*Ph. edulis* (10)	PeGRAS-44 (PH01000054G0400); PeGRAS-45 (PH01000374G0830); PeGRAS-46 (PH01000138G1130); PeGRAS-47 (PH01001761G0380); PeGRAS-48 (PH01000369G0400); PeGRAS-49 (PH01005526G0030); PeGRAS-50 (PH01003146G0100); PeGRAS-51 (PH01004053G0150); PeGRAS-52 (PH01003146G0070); PeGRAS-53 (PH01005978G0070)
	*O. sativa* (11)	OsGRAS-22 (Os04g50060); OsGRAS-16 (Os03g48450); OsGRAS-2 (Os01g62460); OsGRAS-45 (Os11g47890); OsGRAS-44 (Os11g47870); OsGRAS-53 (Os12g38490); OsGRAS-48 (Os11g47920); OsGRAS-47 (Os11g47910); OsGRAS-46 (Os11g47900); OsGRAS-39 (Os11g04400); OsGRAS-50 (Os12g04200)
	*B. distachyon* (13)	(Bradi2g54670.2); (Bradi1g03620.1); (Bradi4g43680.2); (Bradi1g00220.1); (Bradi4g09235.1); (Bradi4g09155.1); (Bradi4g03867.1); (Bradi4g09180.1); (Bradi4g09190.1); (Bradi4g09160.1); (Bradi4g09170.1); (Bradi4g09197.1); (Bradi2g52227.1)
	*A. thaliana* (5)	AtSCL9 (At2g37650); AtSCL31 (At1g07520); AtSCL14 (At1g07530); AtSCL30 (At3g46600); AtSCL11(At5g59450)
AtLAS	*Ph. edulis* (0)	
	*O. sativa* (2)	OsGRAS-7 (Os02g10360); OsMOC1 (Os06g40780)
	*B. distachyon* (2)	(Bradi1g36180.1); (Bradi3g07160.1)
	*A. thaliana* (1)	AtLAS (At1g55580)
Unknown	*Ph. edulis* (6)	PeGRAS-54 (PH01143550G0010); PeGRAS-55 (PH01000254G0530); PeGRAS-56 (PH01004823G0070); PeGRAS-57 (PH01002560G0270); PeGRAS-58 (PH01000356G0990); PeGRAS-59 (PH01004053G0220)
	*O. sativa* (8)	OsGRAS-19 (Os04g35250); OsGRAS-27 (Os05g49930); OsGRAS-1 (Os01g45860); OsGRAS-5 (Os01g67670); OsGRAS-4 (Os01g67650); OsGRAS-43 (Os11g31100); OsGRAS-15 (Os03g40080); OsGRAS-42 (Os11g11600)
	*B. distachyon* (7)	(Bradi1g15123.1); (Bradi5g10320.1); (Bradi2g57940.1); (Bradi4g18390.1); (Bradi1g32070.1); (Bradi1g47900.1); (Bradi2g45117.1)
	*A. thaliana* (0)	

^a^The investigated species included *Ph. edulis*, *O. sativa* subsp. *Indica, B*. distachyon and A. thaliana.

^b^The locus name of gene was shown in brackets.

^c^The number of gene was shown in brackets.
