# Supplementary material for: Comprehensive analysis of multi-tissue transcriptome data and the genome-wide investigation of GRAS family in Phyllostachys edulis
Source: Sci Rep. 2016 Jun 21;6:27640. doi: 10.1038/srep27640 (PMC4914925; doi:10.1038/srep27640)
Supplement: Supplementary Information [file srep27640-s1.doc]

*Supplementary File*

**Comprehensive analysis of multi-tissue transcriptome data and the genome-wide investigation of GRAS family in *Phyllostachys edulis***

**Hansheng Zhao^1*^, Lili Dong^1*^, Huayu Sun^1^, Lichao Li^1^, Yongfeng Lou^1^, Lili Wang^1^, Zuyao Li^2‡^ and Zhimin Gao^1‡^**

^1^State Forestry Administration Key Open Laboratory on the Science and Technology of Bamboo and Rattan, International Center for Bamboo and Rattan, Beijing 100102, China

^2^Jiangxi Agricultural University, Nanchang 330045, China

* These authors contributed equally to this work

‡ Co-corresponding author: jxlizuyao@126.com, and gaozhimin@icbr.ac.cn


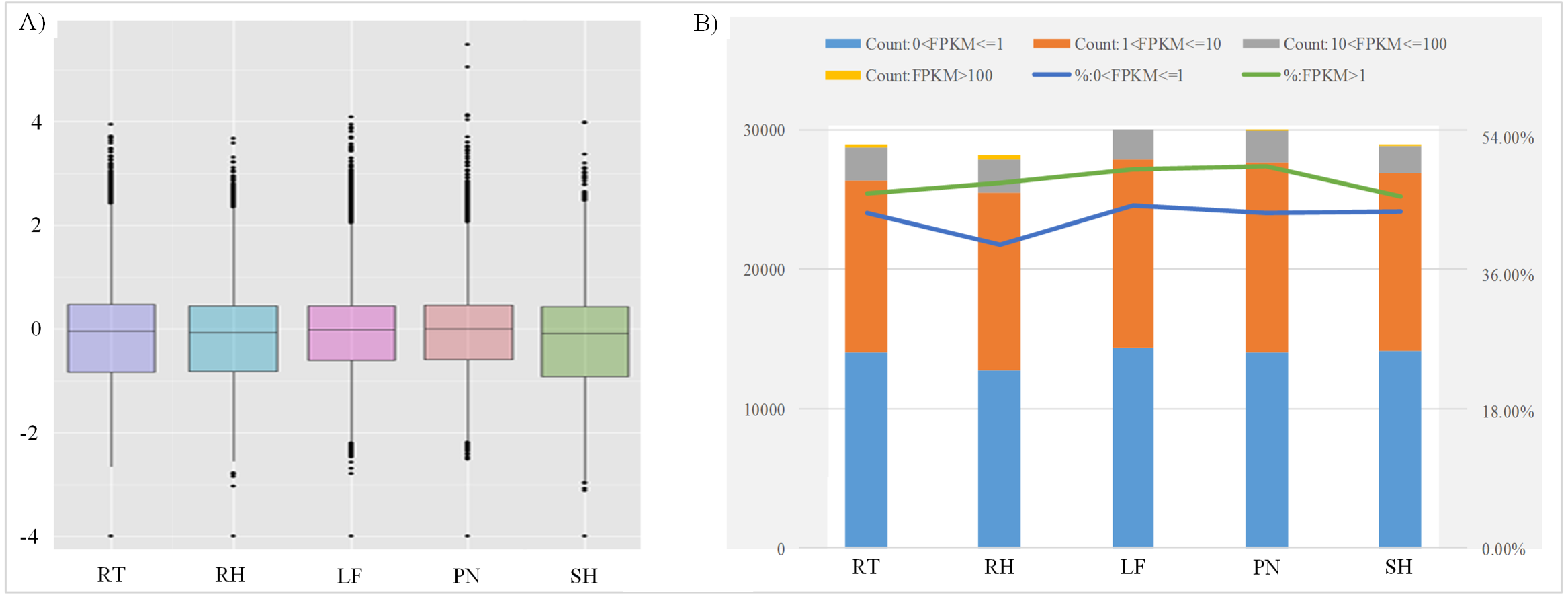


Supplementary Figure S1. Distribution of gene expression values among five tissues. (A) The overview of FPKM distribution among tissues. X-axes: tissues. Y-axis: FPKM based log-transformed, and (B) The different categories based on FPKM value.


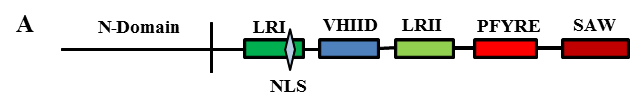


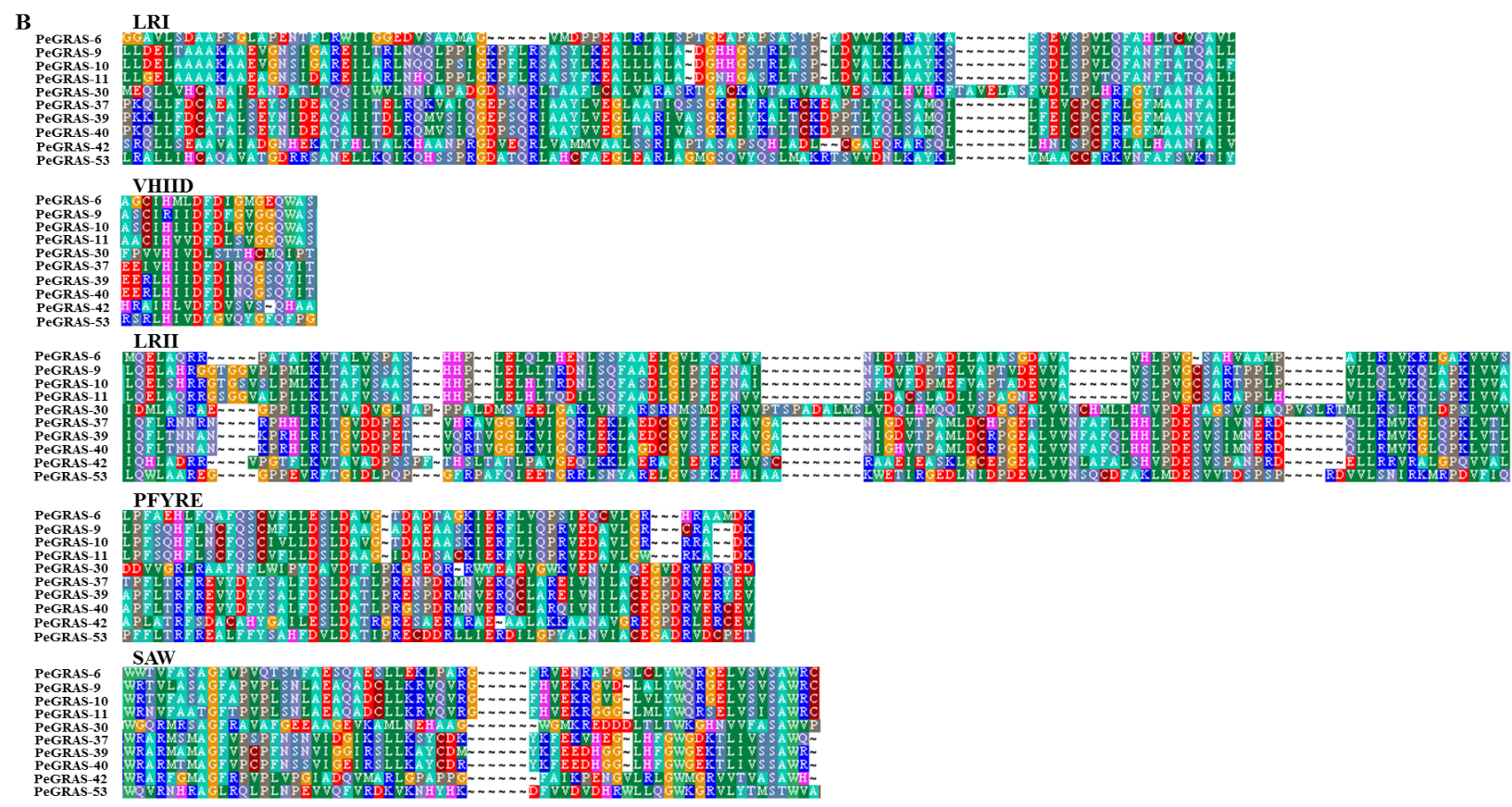


Supplementary Figure S2. The domain structure of GRAS protein. A. Domain structure of GRAS protein. The five conserved motifs in the C-domain contain LRH I, LRH II, VHIID, PFYRE, and SAW. B. Five motifs in ten GRAS proteins from moso bamboo. Ten GRAS proteins: PeGRAS-6, PeGRAS-9, PeGRAS-10, PeGRAS-11, PeGRAS-30, PeGRAS-37, PeGRAS-39, PeGRAS-40, PeGRAS-42, and PeGRAS-53.

Supplementary Figure S3. The information of motifs in 59 GRAS proteins with MEME program

| **Motif** | **E-value** | **Width** | **Sites** | **Sequence logo** | **Regular expression** |
| --- | --- | --- | --- | --- | --- |
| **Motif 1** | 3.7e-435 | 21 | 42 | 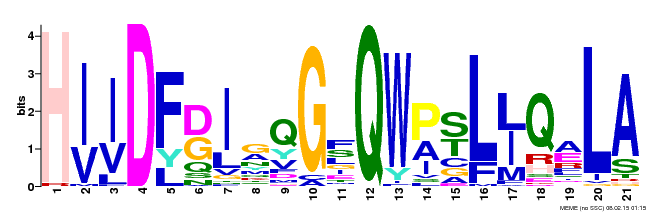 | H[IV][IV]DF[DG]IGQGFQW[PA][ST]L[LI]Q[AE]LA |
| **Motif 2** | 2.2e-403 | 21 | 52 | 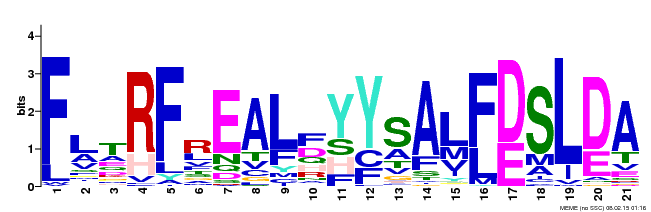 | FLTRFREALFYYSAL[FL][DE]SL[DE]A |
| **Motif 3** | 1.7e-412 | 21 | 51 | 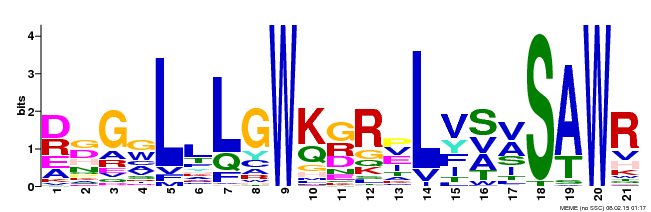 | [DR]xGGLLLGW[KQ][GR]RxLV[SAV][VA]SAWR |
| **Motif 4** | 4.4e-326 | 18 | 39 | 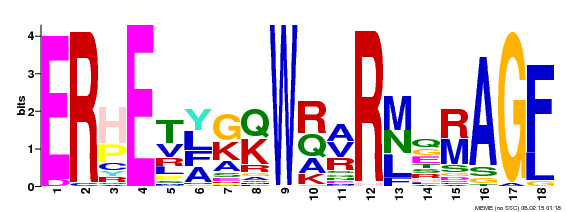 | ER[HP]E[TV][YLAF][GK][QK]W[RQ][AVR]R[MLN]  Q[RM]AG[FL] |
| **Motif 5** | 4.0e-341 | 21 | 52 | 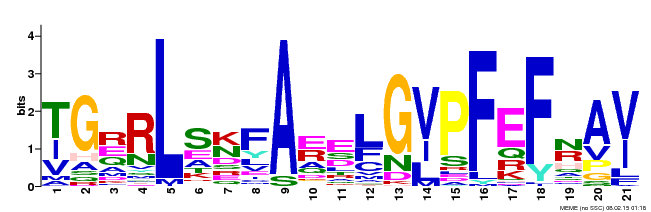 | [TI]GRRLS[KN]FA[ER]ELG[VI]PFEF[NR]A[VI] |
| **Motif 6** | 9.2e-319 | 21 | 52 | 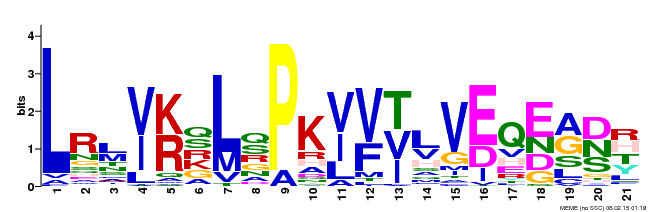 | LRL[VI][KR]xLQPK[VI][VF][TVI]LV[ED]QE[AG][DNS][HR] |
| **Motif 7** | 3.2e-323 | 29 | 29 | 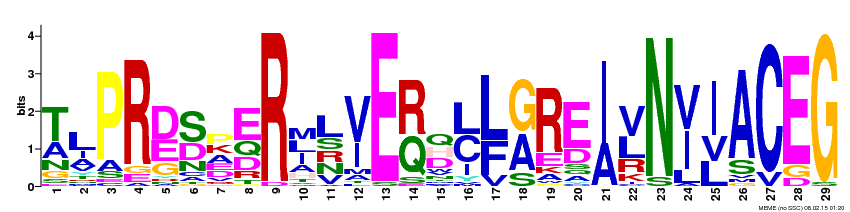 | [TA]LPR[DE][SD]P[EQ]R[ILM]L[VI]E[RQ][QDH][LC][LF][GA]RE[IA][VL]N[VI][ILV]ACEG |
| **Motif 8** | 1.5e-297 | 29 | 27 | 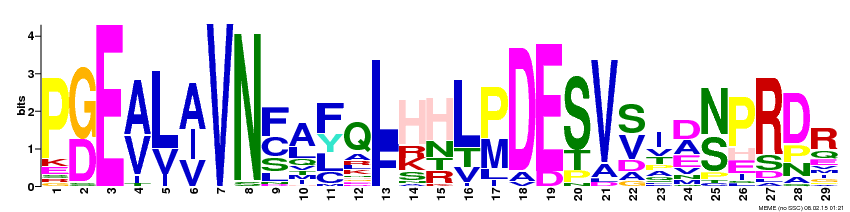 | P[GD]E[AV][LV][AIV]VN[FC]A[FLY]Q[LF]HHL[PM]DESV[SV]I[DA][NS]PRDR |
| **Motif 9** | 3.5e-261 | 16 | 47 | 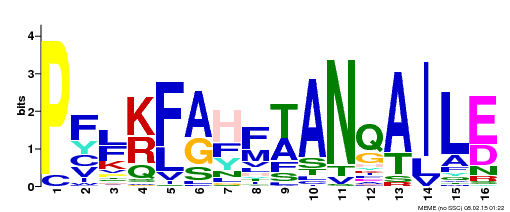 | PF[FL][KR][FL][AG]HF[TA]ANQAIL[ED] |
| **Motif 10** | 3.2e-322 | 29 | 43 | 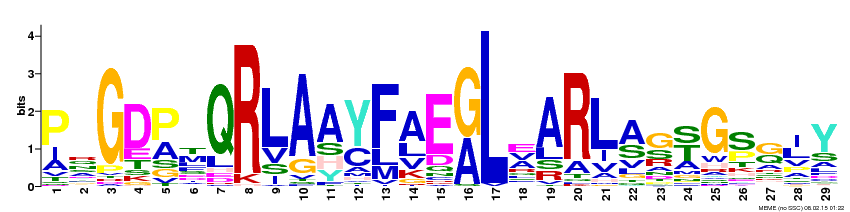 | PxGD[PA]xQRLAA[YC]FAE[GA]LEARL[AS]G[ST]GS[GQ][IL]Y |

The height of a letter indicates its relative frequency at the given position.


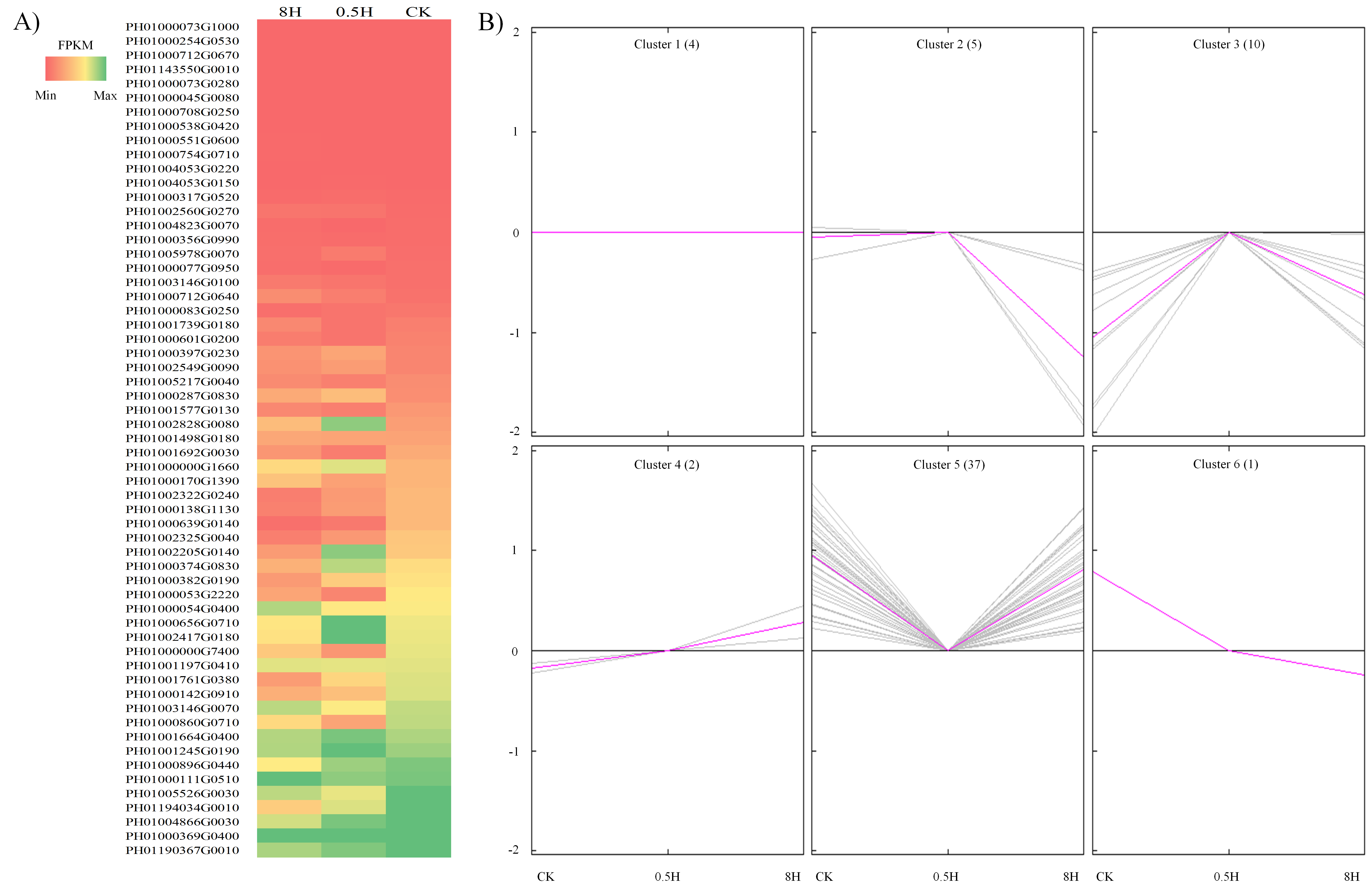


Supplementary Figure S4. The expression profiles of *PeGRAS*s in response to high light. A) The heatmap based on clustered expression value. B) The cluster analysis of expressed genes based on CAST method. The number of genes in each group was showed in brackets. The fitting result in each group was displayed by pink line.

Supplementary Figure S5. Comparative analysis of alignment quality using TopHat with/without the parameters of the expected inner distance and the standard deviation.


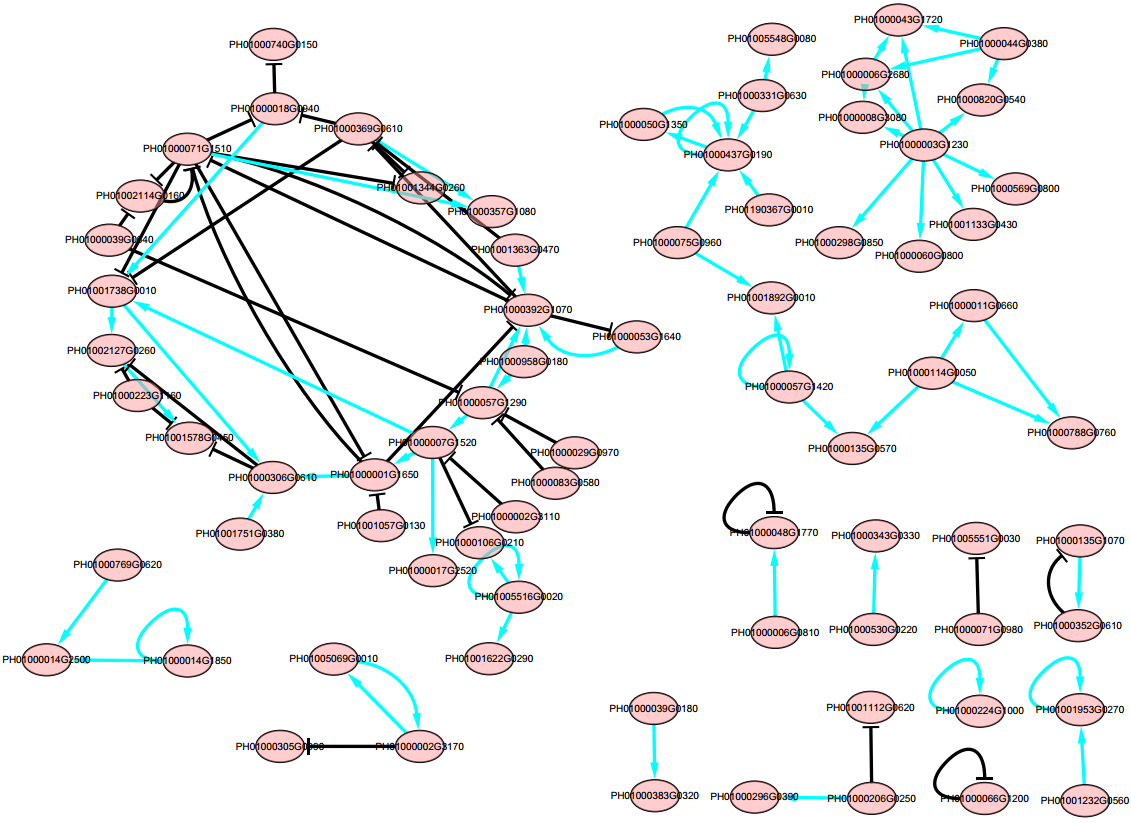


**Figure S6. The TF networks predicted in moso bamboo.** The network was provided by Cytoscape 3.0. Black line represented the regulated relationship of repressed transcript, blue line represented the regulated relationship of activated transcript, and the locus name of moso bamboo was provided in an oval.

| Supplementary Table S1. GO enrichment analysis of based on conservatively biological function for expression gene in within-tissue. | | | | | | | | | | | | |
| --- | --- | --- | --- | --- | --- | --- | --- | --- | --- | --- | --- | --- |
| ID | Pop.total | Pop.term | Study.total | Study.term | Pop.family | Study.family | Nparents | Is.trivial | p | p. adjusted | Name |  |
| GO:0006412 | 13453 | 529 | 7497 | 432 | 3894 | 2095 | 3 | FALSE | 6.28E-47 | 1.28E-43 | translation |  |
| GO:0005575 | 13453 | 2737 | 7497 | 1829 | 13453 | 7497 | 1 | FALSE | 3.99E-40 | 8.09E-37 | cellular_component |  |
| GO:0005198 | 13453 | 464 | 7497 | 388 | 13352 | 7436 | 1 | FALSE | 1.08E-38 | 2.19E-35 | structural molecule activity |  |
| GO:0044281 | 13453 | 713 | 7497 | 505 | 1422 | 782 | 1 | FALSE | 5.93E-34 | 1.20E-30 | small molecule metabolic process |  |
| GO:0009987 | 13453 | 5874 | 7497 | 3400 | 7776 | 4217 | 1 | FALSE | 5.25E-30 | 1.07E-26 | cellular process |  |
| GO:0009056 | 13453 | 489 | 7497 | 374 | 6889 | 3734 | 1 | FALSE | 3.63E-26 | 7.37E-23 | catabolic process |  |
| GO:1901575 | 13453 | 487 | 7497 | 373 | 5935 | 3317 | 2 | FALSE | 3.33E-23 | 6.75E-20 | organic substance catabolic process |  |
| GO:0008270 | 13453 | 803 | 7497 | 488 | 1248 | 631 | 1 | FALSE | 1.36E-22 | 2.76E-19 | zinc ion binding |  |
| GO:0006753 | 13453 | 263 | 7497 | 202 | 1959 | 991 | 3 | FALSE | 7.82E-21 | 1.59E-17 | nucleoside phosphate metabolic process |  |
| GO:1901564 | 13453 | 664 | 7497 | 481 | 5935 | 3318 | 2 | FALSE | 1.08E-20 | 2.20E-17 | organonitrogen compound metabolic process |  |
| GO:0007165 | 13453 | 268 | 7497 | 192 | 1419 | 682 | 4 | FALSE | 3.84E-18 | 7.80E-15 | signal transduction |  |
| GO:0005623 | 13453 | 2050 | 7497 | 1463 | 2737 | 1829 | 1 | FALSE | 7.17E-18 | 1.46E-14 | cell |  |
| GO:0044464 | 13453 | 2050 | 7497 | 1463 | 2737 | 1829 | 2 | FALSE | 7.17E-18 | 1.46E-14 | cell part |  |
| GO:0043226 | 13453 | 1321 | 7497 | 987 | 2737 | 1829 | 1 | FALSE | 1.25E-17 | 2.55E-14 | organelle |  |
| GO:0033036 | 13453 | 156 | 7497 | 125 | 704 | 360 | 1 | FALSE | 3.38E-17 | 6.86E-14 | macromolecule localization |  |
| GO:0046907 | 13453 | 133 | 7497 | 110 | 704 | 360 | 2 | FALSE | 6.35E-17 | 1.29E-13 | intracellular transport |  |
| GO:0019693 | 13453 | 188 | 7497 | 149 | 2003 | 1027 | 3 | FALSE | 8.98E-17 | 1.82E-13 | ribose phosphate metabolic process |  |
| GO:0045184 | 13453 | 134 | 7497 | 109 | 704 | 360 | 2 | FALSE | 1.14E-15 | 2.32E-12 | establishment of protein localization |  |
| GO:0044723 | 13453 | 264 | 7497 | 194 | 696 | 379 | 1 | FALSE | 1.22E-15 | 2.48E-12 | single-organism carbohydrate metabolic process |  |
| GO:0044248 | 13453 | 316 | 7497 | 247 | 5274 | 3032 | 2 | FALSE | 1.52E-15 | 3.08E-12 | cellular catabolic process |  |
| GO:1901265 | 13453 | 3395 | 7497 | 2000 | 6039 | 3291 | 2 | FALSE | 3.59E-15 | 7.29E-12 | nucleoside phosphate binding |  |
| GO:0030163 | 13453 | 136 | 7497 | 118 | 2951 | 1661 | 2 | FALSE | 5.28E-15 | 1.07E-11 | protein catabolic process |  |
| GO:0016817 | 13453 | 949 | 7497 | 649 | 2217 | 1310 | 1 | FALSE | 6.61E-15 | 1.34E-11 | hydrolase activity, acting on acid anhydrides |  |
| GO:0032991 | 13453 | 1168 | 7497 | 872 | 2737 | 1829 | 1 | FALSE | 2.60E-14 | 5.29E-11 | macromolecular complex |  |
| GO:1901135 | 13453 | 287 | 7497 | 220 | 5933 | 3316 | 1 | FALSE | 5.35E-14 | 1.09E-10 | carbohydrate derivative metabolic process |  |
| GO:1901657 | 13453 | 188 | 7497 | 150 | 6460 | 3491 | 2 | FALSE | 7.20E-14 | 1.46E-10 | glycosyl compound metabolic process |  |
| GO:0044699 | 13453 | 1222 | 7497 | 780 | 7776 | 4217 | 1 | FALSE | 9.43E-14 | 1.91E-10 | single-organism process |  |
| GO:0019637 | 13453 | 318 | 7497 | 240 | 5967 | 3346 | 2 | FALSE | 1.33E-13 | 2.69E-10 | organophosphate metabolic process |  |
| GO:0071702 | 13453 | 205 | 7497 | 148 | 704 | 360 | 1 | FALSE | 3.55E-13 | 7.22E-10 | organic substance transport |  |
| GO:0051603 | 13453 | 111 | 7497 | 95 | 465 | 266 | 2 | FALSE | 4.36E-13 | 8.85E-10 | proteolysis involved in cellular protein catabolic process |  |
| GO:0019001 | 13453 | 203 | 7497 | 161 | 2881 | 1622 | 1 | FALSE | 9.40E-13 | 1.91E-09 | guanyl nucleotide binding |  |
| GO:0044237 | 13453 | 5214 | 7497 | 2995 | 7515 | 4116 | 2 | FALSE | 1.59E-12 | 3.23E-09 | cellular metabolic process |  |
| GO:0071704 | 13453 | 5933 | 7497 | 3316 | 6889 | 3734 | 1 | FALSE | 1.70E-12 | 3.45E-09 | organic substance metabolic process |  |
| GO:0055086 | 13453 | 287 | 7497 | 216 | 2376 | 1357 | 2 | FALSE | 8.12E-12 | 1.65E-08 | nucleobase-containing small molecule metabolic process |  |
| GO:0071840 | 13453 | 308 | 7497 | 224 | 7776 | 4217 | 1 | FALSE | 8.19E-12 | 1.66E-08 | cellular component organization or biogenesis |  |
| GO:0005525 | 13453 | 196 | 7497 | 154 | 2865 | 1610 | 3 | FALSE | 1.03E-11 | 2.09E-08 | GTP binding |  |
| GO:0072521 | 13453 | 209 | 7497 | 164 | 2361 | 1348 | 5 | FALSE | 1.06E-11 | 2.15E-08 | purine-containing compound metabolic process |  |
| GO:0032561 | 13453 | 196 | 7497 | 154 | 2872 | 1617 | 2 | FALSE | 1.27E-11 | 2.58E-08 | guanyl ribonucleotide binding |  |
| GO:0030529 | 13453 | 410 | 7497 | 348 | 1902 | 1376 | 2 | FALSE | 1.67E-11 | 3.39E-08 | ribonucleoprotein complex |  |
| GO:0044265 | 13453 | 126 | 7497 | 105 | 4398 | 2449 | 3 | FALSE | 2.43E-11 | 4.93E-08 | cellular macromolecule catabolic process |  |
| GO:0003723 | 13453 | 514 | 7497 | 363 | 2963 | 1711 | 1 | FALSE | 2.79E-11 | 5.66E-08 | RNA binding |  |
| GO:0051649 | 13453 | 179 | 7497 | 129 | 704 | 360 | 2 | FALSE | 4.33E-11 | 8.79E-08 | establishment of localization in cell |  |
| GO:0044257 | 13453 | 111 | 7497 | 95 | 2578 | 1494 | 3 | FALSE | 1.43E-10 | 2.91E-07 | cellular protein catabolic process |  |
| GO:0034655 | 13453 | 162 | 7497 | 127 | 2044 | 1135 | 5 | FALSE | 2.35E-10 | 4.77E-07 | nucleobase-containing compound catabolic process |  |
| GO:0006457 | 13453 | 132 | 7497 | 109 | 2538 | 1461 | 1 | FALSE | 3.25E-10 | 6.60E-07 | protein folding |  |
| GO:0044238 | 13453 | 5818 | 7497 | 3245 | 6889 | 3734 | 1 | FALSE | 6.69E-10 | 1.36E-06 | primary metabolic process |  |
| GO:0044270 | 13453 | 165 | 7497 | 130 | 2302 | 1323 | 2 | FALSE | 2.08E-09 | 4.23E-06 | cellular nitrogen compound catabolic process |  |
| GO:0023052 | 13453 | 268 | 7497 | 192 | 7776 | 4217 | 1 | FALSE | 2.13E-09 | 4.32E-06 | signaling |  |
| GO:0006082 | 13453 | 385 | 7497 | 270 | 6099 | 3413 | 3 | FALSE | 2.73E-09 | 5.55E-06 | organic acid metabolic process |  |
| GO:0005737 | 13453 | 892 | 7497 | 702 | 1864 | 1349 | 1 | FALSE | 2.77E-09 | 5.63E-06 | cytoplasm |  |
| GO:0006520 | 13453 | 308 | 7497 | 221 | 5904 | 3306 | 3 | FALSE | 3.75E-09 | 7.61E-06 | cellular amino acid metabolic process |  |
| GO:0019439 | 13453 | 167 | 7497 | 131 | 2285 | 1319 | 2 | FALSE | 4.47E-09 | 9.08E-06 | aromatic compound catabolic process |  |
| GO:0016787 | 13453 | 2217 | 7497 | 1310 | 6400 | 3467 | 1 | FALSE | 4.97E-09 | 1.01E-05 | hydrolase activity |  |
| GO:0016614 | 13453 | 133 | 7497 | 87 | 743 | 318 | 1 | FALSE | 5.85E-09 | 1.19E-05 | oxidoreductase activity, acting on CH-OH group of donors |  |
| GO:0046700 | 13453 | 167 | 7497 | 130 | 2271 | 1303 | 2 | FALSE | 7.40E-09 | 1.50E-05 | heterocycle catabolic process |  |
| GO:0051082 | 13453 | 70 | 7497 | 61 | 3820 | 2150 | 1 | FALSE | 2.14E-08 | 4.35E-05 | unfolded protein binding |  |
| GO:1901361 | 13453 | 168 | 7497 | 131 | 2460 | 1437 | 2 | FALSE | 2.76E-08 | 5.60E-05 | organic cyclic compound catabolic process |  |
| GO:0044444 | 13453 | 694 | 7497 | 552 | 1864 | 1349 | 2 | FALSE | 4.42E-08 | 8.98E-05 | cytoplasmic part |  |
| GO:0036094 | 13453 | 3424 | 7497 | 2022 | 10284 | 5691 | 1 | FALSE | 4.60E-08 | 9.33E-05 | small molecule binding |  |
| GO:0009058 | 13453 | 2292 | 7497 | 1345 | 6889 | 3734 | 1 | FALSE | 7.47E-08 | 1.52E-04 | biosynthetic process |  |
| GO:0043228 | 13453 | 675 | 7497 | 546 | 1321 | 987 | 1 | FALSE | 8.61E-08 | 1.75E-04 | non-membrane-bounded organelle |  |
| GO:0043232 | 13453 | 675 | 7497 | 546 | 1321 | 987 | 2 | FALSE | 8.61E-08 | 1.75E-04 | intracellular non-membrane-bounded organelle |  |
| GO:0009057 | 13453 | 191 | 7497 | 141 | 4854 | 2699 | 2 | FALSE | 8.88E-08 | 1.80E-04 | macromolecule catabolic process |  |
| GO:0044422 | 13453 | 531 | 7497 | 405 | 2737 | 1829 | 2 | FALSE | 9.52E-08 | 1.93E-04 | organelle part |  |
| GO:0006486 | 13453 | 32 | 7497 | 29 | 1752 | 817 | 3 | FALSE | 1.62E-07 | 3.29E-04 | protein glycosylation |  |
| GO:0008026 | 13453 | 131 | 7497 | 106 | 270 | 177 | 2 | FALSE | 1.73E-07 | 3.51E-04 | ATP-dependent helicase activity |  |
| GO:0043413 | 13453 | 32 | 7497 | 29 | 1801 | 856 | 2 | FALSE | 2.73E-07 | 5.54E-04 | macromolecule glycosylation |  |
| GO:1901566 | 13453 | 292 | 7497 | 214 | 2534 | 1518 | 2 | FALSE | 2.80E-07 | 5.69E-04 | organonitrogen compound biosynthetic process |  |
| GO:0044267 | 13453 | 2538 | 7497 | 1461 | 4534 | 2461 | 2 | FALSE | 3.20E-07 | 6.50E-04 | cellular protein metabolic process |  |
| GO:0008237 | 13453 | 56 | 7497 | 47 | 427 | 228 | 1 | FALSE | 3.27E-07 | 6.63E-04 | metallopeptidase activity |  |
| GO:0044763 | 13453 | 1143 | 7497 | 736 | 5952 | 3444 | 2 | FALSE | 3.32E-07 | 6.74E-04 | single-organism cellular process |  |
| GO:0003676 | 13453 | 2963 | 7497 | 1711 | 6039 | 3291 | 2 | FALSE | 3.64E-07 | 7.40E-04 | nucleic acid binding |  |
| GO:0070003 | 13453 | 39 | 7497 | 35 | 427 | 228 | 1 | FALSE | 4.67E-07 | 9.48E-04 | threonine-type peptidase activity |  |
| GO:0004298 | 13453 | 39 | 7497 | 35 | 345 | 187 | 2 | FALSE | 5.96E-07 | 0.001209189 | threonine-type endopeptidase activity |  |
| GO:0004386 | 13453 | 210 | 7497 | 172 | 899 | 619 | 1 | FALSE | 1.08E-06 | 0.002186621 | helicase activity |  |
| GO:0016790 | 13453 | 63 | 7497 | 53 | 441 | 254 | 1 | FALSE | 1.44E-06 | 0.00291502 | thiolester hydrolase activity |  |
| GO:0070727 | 13453 | 127 | 7497 | 106 | 209 | 149 | 2 | FALSE | 1.57E-06 | 0.003189698 | cellular macromolecule localization |  |
| GO:0044711 | 13453 | 222 | 7497 | 156 | 3255 | 1812 | 2 | FALSE | 2.78E-06 | 0.005648029 | single-organism biosynthetic process |  |
| GO:0048475 | 13453 | 43 | 7497 | 38 | 877 | 495 | 1 | FALSE | 4.17E-06 | 0.008465067 | coated membrane |  |
| GO:0034660 | 13453 | 118 | 7497 | 81 | 1360 | 668 | 1 | FALSE | 5.86E-06 | 0.011889785 | ncRNA metabolic process |  |
| GO:0042623 | 13453 | 270 | 7497 | 177 | 376 | 220 | 1 | FALSE | 8.87E-06 | 0.018002305 | ATPase activity, coupled |  |
| GO:0023051 | 13453 | 46 | 7497 | 36 | 1346 | 629 | 2 | FALSE | 9.02E-06 | 0.018303069 | regulation of signaling |  |
| GO:0051186 | 13453 | 88 | 7497 | 70 | 5214 | 2995 | 1 | FALSE | 9.12E-06 | 0.018522051 | cofactor metabolic process |  |
| GO:1901137 | 13453 | 90 | 7497 | 73 | 2437 | 1457 | 2 | FALSE | 9.32E-06 | 0.018911955 | carbohydrate derivative biosynthetic process |  |
| GO:0019205 | 13453 | 32 | 7497 | 27 | 1686 | 786 | 1 | FALSE | 9.73E-06 | 0.019752323 | nucleobase-containing compound kinase activity |  |
| GO:0010646 | 13453 | 46 | 7497 | 36 | 1297 | 612 | 2 | FALSE | 1.18E-05 | 0.023866286 | regulation of cell communication |  |
| GO:0016071 | 13453 | 59 | 7497 | 45 | 1360 | 668 | 1 | FALSE | 1.25E-05 | 0.025358467 | mRNA metabolic process |  |
| GO:0050896 | 13453 | 607 | 7497 | 379 | 7776 | 4217 | 1 | FALSE | 1.28E-05 | 0.025938003 | response to stimulus |  |
| GO:0016570 | 13453 | 22 | 7497 | 20 | 1752 | 817 | 2 | FALSE | 1.52E-05 | 0.030891385 | histone modification |  |
| GO:0009163 | 13453 | 37 | 7497 | 32 | 1577 | 839 | 5 | FALSE | 1.64E-05 | 0.033290707 | nucleoside biosynthetic process |  |
| GO:0009100 | 13453 | 32 | 7497 | 29 | 4771 | 2644 | 3 | FALSE | 1.74E-05 | 0.035348693 | glycoprotein metabolic process |  |
| GO:0048583 | 13453 | 46 | 7497 | 36 | 1637 | 782 | 2 | FALSE | 1.78E-05 | 0.03622336 | regulation of response to stimulus |  |
| GO:0005622 | 13453 | 1999 | 7497 | 1441 | 2050 | 1463 | 1 | FALSE | 1.81E-05 | 0.036767806 | intracellular |  |
| GO:0005840 | 13453 | 383 | 7497 | 326 | 1006 | 788 | 3 | FALSE | 2.13E-05 | 0.043316915 | ribosome |  |
| GO:0009451 | 13453 | 40 | 7497 | 32 | 3095 | 1471 | 2 | FALSE | 2.31E-05 | 0.046915177 | RNA modification |  |

| Supplementary Table S2. GO enrichment based on conservatively biological function for expression gene among Cluster 1 | | | | | | | | | | | | |
| --- | --- | --- | --- | --- | --- | --- | --- | --- | --- | --- | --- | --- |
| ID | Pop.total | Pop.term | Study.total | Study.term | Pop.family | Study.family | Nparents | Is.trivial | p | p. adjusted | Name |  |
| GO:0006412 | 13453 | 529 | 2131 | 262 | 3894 | 726 | 3 | FALSE | 4.48E-70 | 6.77E-67 | translation |  |
| GO:0005198 | 13453 | 464 | 2131 | 218 | 13352 | 2113 | 1 | FALSE | 2.25E-58 | 3.40E-55 | structural molecule activity |  |
| GO:0032991 | 13453 | 1168 | 2131 | 412 | 2737 | 643 | 1 | FALSE | 5.58E-36 | 8.44E-33 | macromolecular complex |  |
| GO:0005623 | 13453 | 2050 | 2131 | 591 | 2737 | 643 | 1 | FALSE | 3.99E-35 | 6.03E-32 | cell |  |
| GO:0044464 | 13453 | 2050 | 2131 | 591 | 2737 | 643 | 2 | FALSE | 3.99E-35 | 6.03E-32 | cell part |  |
| GO:0009058 | 13453 | 2292 | 2131 | 532 | 6889 | 1069 | 1 | FALSE | 3.65E-34 | 5.52E-31 | biosynthetic process |  |
| GO:0030529 | 13453 | 410 | 2131 | 224 | 1902 | 571 | 2 | FALSE | 1.46E-32 | 2.21E-29 | ribonucleoprotein complex |  |
| GO:0005575 | 13453 | 2737 | 2131 | 643 | 13453 | 2131 | 1 | FALSE | 2.37E-32 | 3.59E-29 | cellular_component |  |
| GO:0009987 | 13453 | 5874 | 2131 | 1040 | 7776 | 1183 | 1 | FALSE | 2.00E-30 | 3.03E-27 | cellular process |  |
| GO:0043226 | 13453 | 1321 | 2131 | 432 | 2737 | 643 | 1 | FALSE | 1.85E-28 | 2.79E-25 | organelle |  |
| GO:1901576 | 13453 | 2244 | 2131 | 526 | 5980 | 1015 | 2 | FALSE | 1.63E-24 | 2.46E-21 | organic substance biosynthetic process |  |
| GO:0044238 | 13453 | 5818 | 2131 | 1003 | 6889 | 1069 | 1 | FALSE | 5.51E-24 | 8.34E-21 | primary metabolic process |  |
| GO:0005840 | 13453 | 383 | 2131 | 211 | 1006 | 363 | 3 | FALSE | 9.36E-23 | 1.41E-19 | ribosome |  |
| GO:0009059 | 13453 | 1805 | 2131 | 456 | 4996 | 914 | 2 | FALSE | 2.66E-21 | 4.02E-18 | macromolecule biosynthetic process |  |
| GO:0071704 | 13453 | 5933 | 2131 | 1010 | 6889 | 1069 | 1 | FALSE | 3.08E-21 | 4.66E-18 | organic substance metabolic process |  |
| GO:0004386 | 13453 | 210 | 2131 | 117 | 899 | 261 | 1 | FALSE | 5.62E-21 | 8.50E-18 | helicase activity |  |
| GO:0044249 | 13453 | 2216 | 2131 | 524 | 5284 | 942 | 2 | FALSE | 7.23E-21 | 1.09E-17 | cellular biosynthetic process |  |
| GO:0016787 | 13453 | 2217 | 2131 | 449 | 6400 | 933 | 1 | FALSE | 2.64E-20 | 3.99E-17 | hydrolase activity |  |
| GO:0003676 | 13453 | 2963 | 2131 | 647 | 6039 | 1049 | 2 | FALSE | 1.30E-19 | 1.97E-16 | nucleic acid binding |  |
| GO:0016817 | 13453 | 949 | 2131 | 274 | 2217 | 449 | 1 | FALSE | 2.61E-18 | 3.94E-15 | hydrolase activity, acting on acid anhydrides |  |
| GO:0044237 | 13453 | 5214 | 2131 | 935 | 7515 | 1172 | 2 | FALSE | 2.79E-18 | 4.22E-15 | cellular metabolic process |  |
| GO:0034645 | 13453 | 1802 | 2131 | 456 | 4590 | 872 | 3 | FALSE | 2.93E-18 | 4.43E-15 | cellular macromolecule biosynthetic process |  |
| GO:0010467 | 13453 | 1768 | 2131 | 439 | 4561 | 844 | 1 | FALSE | 3.30E-18 | 4.99E-15 | gene expression |  |
| GO:0003723 | 13453 | 514 | 2131 | 187 | 2963 | 647 | 1 | FALSE | 4.19E-17 | 6.34E-14 | RNA binding |  |
| GO:0043228 | 13453 | 675 | 2131 | 290 | 1321 | 432 | 1 | FALSE | 2.15E-16 | 3.26E-13 | non-membrane-bounded organelle |  |
| GO:0043232 | 13453 | 675 | 2131 | 290 | 1321 | 432 | 2 | FALSE | 2.15E-16 | 3.26E-13 | intracellular non-membrane-bounded organelle |  |
| GO:0003735 | 13453 | 383 | 2131 | 211 | 464 | 218 | 1 | FALSE | 5.47E-16 | 8.27E-13 | structural constituent of ribosome |  |
| GO:0009451 | 13453 | 40 | 2131 | 25 | 3095 | 394 | 2 | FALSE | 1.35E-13 | 2.04E-10 | RNA modification |  |
| GO:0044281 | 13453 | 713 | 2131 | 120 | 1422 | 156 | 1 | FALSE | 3.77E-13 | 5.70E-10 | small molecule metabolic process |  |
| GO:0008026 | 13453 | 131 | 2131 | 77 | 270 | 102 | 2 | FALSE | 2.64E-12 | 3.99E-09 | ATP-dependent helicase activity |  |
| GO:0044444 | 13453 | 694 | 2131 | 273 | 1864 | 562 | 2 | FALSE | 2.79E-11 | 4.22E-08 | cytoplasmic part |  |
| GO:0016779 | 13453 | 173 | 2131 | 48 | 1873 | 202 | 1 | FALSE | 3.61E-11 | 5.46E-08 | nucleotidyltransferase activity |  |
| GO:0044260 | 13453 | 4177 | 2131 | 805 | 5598 | 974 | 2 | FALSE | 3.96E-11 | 5.98E-08 | cellular macromolecule metabolic process |  |
| GO:0051603 | 13453 | 111 | 2131 | 42 | 465 | 80 | 2 | FALSE | 6.30E-10 | 9.53E-07 | proteolysis involved in cellular protein catabolic process |  |
| GO:0043170 | 13453 | 4561 | 2131 | 844 | 5933 | 1010 | 1 | FALSE | 7.48E-09 | 1.13E-05 | macromolecule metabolic process |  |
| GO:0005737 | 13453 | 892 | 2131 | 325 | 1864 | 562 | 1 | FALSE | 9.64E-09 | 1.46E-05 | cytoplasm |  |
| GO:0044391 | 13453 | 65 | 2131 | 45 | 1047 | 371 | 4 | FALSE | 1.07E-08 | 1.62E-05 | ribosomal subunit |  |
| GO:0036094 | 13453 | 3424 | 2131 | 645 | 10284 | 1639 | 1 | FALSE | 1.14E-08 | 1.72E-05 | small molecule binding |  |
| GO:0043168 | 13453 | 3148 | 2131 | 532 | 4736 | 707 | 1 | FALSE | 2.85E-08 | 4.31E-05 | anion binding |  |
| GO:0042623 | 13453 | 270 | 2131 | 102 | 376 | 113 | 1 | FALSE | 3.12E-08 | 4.71E-05 | ATPase activity, coupled |  |
| GO:0033554 | 13453 | 130 | 2131 | 36 | 562 | 73 | 2 | FALSE | 1.12E-07 | 1.69E-04 | cellular response to stress |  |
| GO:0009123 | 13453 | 37 | 2131 | 17 | 263 | 38 | 1 | FALSE | 3.51E-07 | 5.30E-04 | nucleoside monophosphate metabolic process |  |
| GO:0008270 | 13453 | 803 | 2131 | 115 | 1248 | 139 | 1 | FALSE | 3.92E-07 | 5.93E-04 | zinc ion binding |  |
| GO:0070003 | 13453 | 39 | 2131 | 18 | 427 | 62 | 1 | FALSE | 4.75E-07 | 7.19E-04 | threonine-type peptidase activity |  |
| GO:0016741 | 13453 | 82 | 2131 | 27 | 2169 | 267 | 1 | FALSE | 5.02E-07 | 7.59E-04 | transferase activity, transferring one-carbon groups |  |
| GO:0006259 | 13453 | 376 | 2131 | 110 | 4177 | 805 | 2 | FALSE | 6.24E-07 | 9.44E-04 | DNA metabolic process |  |
| GO:0006928 | 13453 | 89 | 2131 | 33 | 1143 | 189 | 1 | FALSE | 8.04E-07 | 0.001215148 | cellular component movement |  |
| GO:1901363 | 13453 | 6039 | 2131 | 1049 | 10284 | 1639 | 1 | FALSE | 1.09E-06 | 0.00165305 | heterocyclic compound binding |  |
| GO:0097159 | 13453 | 6039 | 2131 | 1049 | 10284 | 1639 | 1 | FALSE | 1.09E-06 | 0.00165305 | organic cyclic compound binding |  |
| GO:0004298 | 13453 | 39 | 2131 | 18 | 345 | 54 | 2 | FALSE | 1.18E-06 | 0.001778435 | threonine-type endopeptidase activity |  |
| GO:0044265 | 13453 | 126 | 2131 | 46 | 4398 | 822 | 3 | FALSE | 1.34E-06 | 0.002029427 | cellular macromolecule catabolic process |  |
| GO:0007017 | 13453 | 114 | 2131 | 38 | 1143 | 189 | 1 | FALSE | 2.42E-06 | 0.003653657 | microtubule-based process |  |
| GO:0042555 | 13453 | 9 | 2131 | 9 | 752 | 185 | 1 | FALSE | 2.84E-06 | 0.004297047 | MCM complex |  |
| GO:0032259 | 13453 | 25 | 2131 | 14 | 6889 | 1069 | 1 | FALSE | 3.58E-06 | 0.005406357 | methylation |  |
| GO:0043414 | 13453 | 19 | 2131 | 13 | 4182 | 806 | 3 | FALSE | 3.97E-06 | 0.005999579 | macromolecule methylation |  |
| GO:0030163 | 13453 | 136 | 2131 | 47 | 2951 | 549 | 2 | FALSE | 4.37E-06 | 0.006607189 | protein catabolic process |  |
| GO:0006807 | 13453 | 2351 | 2131 | 429 | 6889 | 1069 | 1 | FALSE | 4.78E-06 | 0.007222466 | nitrogen compound metabolic process |  |
| GO:0044257 | 13453 | 111 | 2131 | 42 | 2578 | 515 | 3 | FALSE | 6.44E-06 | 0.009738268 | cellular protein catabolic process |  |
| GO:0044424 | 13453 | 1864 | 2131 | 562 | 2050 | 591 | 2 | FALSE | 8.32E-06 | 0.012577613 | intracellular part |  |
| GO:0005852 | 13453 | 23 | 2131 | 18 | 1241 | 408 | 2 | FALSE | 8.70E-06 | 0.013150929 | eukaryotic translation initiation factor 3 complex |  |
| GO:0040029 | 13453 | 13 | 2131 | 8 | 1018 | 109 | 1 | FALSE | 1.10E-05 | 0.016685254 | regulation of gene expression, epigenetic |  |
| GO:0016570 | 13453 | 22 | 2131 | 10 | 1752 | 167 | 2 | FALSE | 1.12E-05 | 0.016945863 | histone modification |  |
| GO:0006270 | 13453 | 9 | 2131 | 9 | 376 | 110 | 2 | FALSE | 1.24E-05 | 0.018678935 | DNA replication initiation |  |
| GO:0034660 | 13453 | 118 | 2131 | 38 | 1360 | 229 | 1 | FALSE | 1.27E-05 | 0.019132123 | ncRNA metabolic process |  |
| GO:0016875 | 13453 | 84 | 2131 | 30 | 259 | 51 | 1 | FALSE | 1.27E-05 | 0.019203532 | ligase activity, forming carbon-oxygen bonds |  |
| GO:0046040 | 13453 | 12 | 2131 | 8 | 182 | 25 | 2 | FALSE | 1.45E-05 | 0.021984277 | IMP metabolic process |  |
| GO:0016853 | 13453 | 137 | 2131 | 39 | 6400 | 933 | 1 | FALSE | 1.67E-05 | 0.025230691 | isomerase activity |  |
| GO:0009165 | 13453 | 79 | 2131 | 23 | 263 | 38 | 2 | FALSE | 2.32E-05 | 0.035102312 | nucleotide biosynthetic process |  |

| Supplementary Table S3. GO enrichment based on conservatively biological function for expression gene among Cluster 2 | | | | | | | | | | | | |
| --- | --- | --- | --- | --- | --- | --- | --- | --- | --- | --- | --- | --- |
| ID | Pop.total | Pop.term | Study.total | Study.term | Pop.family | Study.family | Nparents | Is.trivial | p | p. adjusted | Name |  |
| GO:0009579 | 13453 | 31 | 1068 | 18 | 1864 | 113 | 1 | FALSE | 3.42E-15 | 3.20E-12 | thylakoid |  |
| GO:0044436 | 13453 | 31 | 1068 | 18 | 1864 | 113 | 2 | FALSE | 3.42E-15 | 3.20E-12 | thylakoid part |  |
| GO:0019219 | 13453 | 1033 | 1068 | 116 | 2087 | 147 | 4 | FALSE | 2.89E-14 | 2.70E-11 | regulation of nucleobase-containing compound metabolic process |  |
| GO:0051171 | 13453 | 1033 | 1068 | 116 | 2440 | 166 | 2 | FALSE | 1.06E-13 | 9.98E-11 | regulation of nitrogen compound metabolic process |  |
| GO:0034357 | 13453 | 31 | 1068 | 18 | 877 | 73 | 3 | FALSE | 4.06E-13 | 3.80E-10 | photosynthetic membrane |  |
| GO:0009521 | 13453 | 25 | 1068 | 15 | 995 | 65 | 4 | FALSE | 6.50E-13 | 6.09E-10 | photosystem |  |
| GO:0003677 | 13453 | 1720 | 1068 | 172 | 2963 | 216 | 1 | FALSE | 3.18E-12 | 2.98E-09 | DNA binding |  |
| GO:0015979 | 13453 | 25 | 1068 | 15 | 5214 | 403 | 1 | FALSE | 2.60E-11 | 2.44E-08 | photosynthesis |  |
| GO:0034654 | 13453 | 1185 | 1068 | 120 | 2171 | 152 | 5 | FALSE | 8.31E-11 | 7.79E-08 | nucleobase-containing compound biosynthetic process |  |
| GO:0032774 | 13453 | 1094 | 1068 | 116 | 2082 | 151 | 3 | FALSE | 1.62E-10 | 1.52E-07 | RNA biosynthetic process |  |
| GO:0006351 | 13453 | 1090 | 1068 | 116 | 1951 | 146 | 3 | FALSE | 5.25E-10 | 4.92E-07 | transcription, DNA-dependent |  |
| GO:1901362 | 13453 | 1302 | 1068 | 129 | 2907 | 197 | 2 | FALSE | 1.15E-09 | 1.08E-06 | organic cyclic compound biosynthetic process |  |
| GO:0019438 | 13453 | 1272 | 1068 | 126 | 2879 | 194 | 2 | FALSE | 1.37E-09 | 1.28E-06 | aromatic compound biosynthetic process |  |
| GO:0009654 | 13453 | 22 | 1068 | 13 | 377 | 36 | 5 | FALSE | 1.64E-09 | 1.54E-06 | photosystem II oxygen evolving complex |  |
| GO:0019898 | 13453 | 23 | 1068 | 13 | 357 | 33 | 1 | FALSE | 1.90E-09 | 1.78E-06 | extrinsic to membrane |  |
| GO:0044271 | 13453 | 1268 | 1068 | 124 | 2884 | 192 | 2 | FALSE | 2.19E-09 | 2.05E-06 | cellular nitrogen compound biosynthetic process |  |
| GO:0018130 | 13453 | 1266 | 1068 | 124 | 2871 | 193 | 2 | FALSE | 4.43E-09 | 4.15E-06 | heterocycle biosynthetic process |  |
| GO:0010556 | 13453 | 1009 | 1068 | 112 | 1855 | 146 | 3 | FALSE | 5.47E-09 | 5.12E-06 | regulation of macromolecule biosynthetic process |  |
| GO:2000112 | 13453 | 1009 | 1068 | 112 | 1802 | 143 | 3 | FALSE | 5.72E-09 | 5.36E-06 | regulation of cellular macromolecule biosynthetic process |  |
| GO:1990204 | 13453 | 36 | 1068 | 13 | 2064 | 124 | 3 | FALSE | 5.07E-08 | 4.75E-05 | oxidoreductase complex |  |
| GO:0010468 | 13453 | 1018 | 1068 | 112 | 1809 | 146 | 2 | FALSE | 7.21E-08 | 6.76E-05 | regulation of gene expression |  |
| GO:0009889 | 13453 | 1009 | 1068 | 112 | 2422 | 188 | 2 | FALSE | 2.00E-07 | 1.87E-04 | regulation of biosynthetic process |  |
| GO:0051252 | 13453 | 1000 | 1068 | 112 | 1451 | 128 | 3 | FALSE | 2.96E-07 | 2.77E-04 | regulation of RNA metabolic process |  |
| GO:0031326 | 13453 | 1009 | 1068 | 112 | 2282 | 180 | 3 | FALSE | 3.24E-07 | 3.03E-04 | regulation of cellular biosynthetic process |  |
| GO:0016301 | 13453 | 1686 | 1068 | 146 | 1873 | 147 | 1 | FALSE | 1.85E-06 | 0.001737353 | kinase activity |  |
| GO:0043169 | 13453 | 1796 | 1068 | 193 | 4736 | 394 | 1 | FALSE | 1.96E-06 | 0.001834883 | cation binding |  |
| GO:0031323 | 13453 | 1076 | 1068 | 120 | 5385 | 413 | 3 | FALSE | 2.75E-06 | 0.002574311 | regulation of cellular metabolic process |  |
| GO:0001071 | 13453 | 635 | 1068 | 83 | 13352 | 1064 | 1 | FALSE | 4.12E-06 | 0.003856495 | nucleic acid binding transcription factor activity |  |
| GO:0055114 | 13453 | 752 | 1068 | 82 | 1422 | 113 | 1 | FALSE | 6.81E-06 | 0.006383056 | oxidation-reduction process |  |
| GO:0080090 | 13453 | 1081 | 1068 | 119 | 5827 | 451 | 2 | FALSE | 1.21E-05 | 0.011317972 | regulation of primary metabolic process |  |
| GO:0060255 | 13453 | 1059 | 1068 | 115 | 4601 | 359 | 2 | FALSE | 2.82E-05 | 0.026458675 | regulation of macromolecule metabolic process |  |
| GO:0050794 | 13453 | 1280 | 1068 | 130 | 5928 | 440 | 2 | FALSE | 2.91E-05 | 0.02722176 | regulation of cellular process |  |

| Supplementary Table S4. GO enrichment based on conservatively biological function for expression gene among Cluster 3 | | | | | | | | | | | | |
| --- | --- | --- | --- | --- | --- | --- | --- | --- | --- | --- | --- | --- |
| ID | Pop.total | Pop.term | Study.total | Study.term | Pop.family | Study.family | Nparents | Is.trivial | p | p. adjusted | Name |  |
| GO:1901575 | 13453 | 487 | 536 | 58 | 5935 | 236 | 2 | FALSE | 5.07E-15 | 4.67E-12 | organic substance catabolic process |  |
| GO:0009056 | 13453 | 489 | 536 | 58 | 6889 | 287 | 1 | FALSE | 9.56E-14 | 8.80E-11 | catabolic process |  |
| GO:0030117 | 13453 | 43 | 536 | 20 | 1442 | 106 | 4 | FALSE | 8.70E-13 | 8.01E-10 | membrane coat |  |
| GO:0034655 | 13453 | 162 | 536 | 24 | 2044 | 59 | 5 | FALSE | 1.43E-12 | 1.32E-09 | nucleobase-containing compound catabolic process |  |
| GO:0072521 | 13453 | 209 | 536 | 27 | 2361 | 70 | 5 | FALSE | 4.61E-12 | 4.25E-09 | purine-containing compound metabolic process |  |
| GO:0048475 | 13453 | 43 | 536 | 20 | 877 | 75 | 1 | FALSE | 7.44E-12 | 6.85E-09 | coated membrane |  |
| GO:0005575 | 13453 | 2737 | 536 | 173 | 13453 | 536 | 1 | FALSE | 2.35E-11 | 2.17E-08 | cellular_component |  |
| GO:1901135 | 13453 | 287 | 536 | 38 | 5933 | 236 | 1 | FALSE | 2.37E-11 | 2.18E-08 | carbohydrate derivative metabolic process |  |
| GO:0016023 | 13453 | 23 | 536 | 12 | 730 | 38 | 3 | FALSE | 5.76E-11 | 5.30E-08 | cytoplasmic membrane-bounded vesicle |  |
| GO:0046700 | 13453 | 167 | 536 | 24 | 2271 | 75 | 2 | FALSE | 1.25E-10 | 1.16E-07 | heterocycle catabolic process |  |
| GO:0044270 | 13453 | 165 | 536 | 24 | 2302 | 77 | 2 | FALSE | 1.36E-10 | 1.25E-07 | cellular nitrogen compound catabolic process |  |
| GO:0019439 | 13453 | 167 | 536 | 24 | 2285 | 78 | 2 | FALSE | 2.80E-10 | 2.58E-07 | aromatic compound catabolic process |  |
| GO:0031982 | 13453 | 23 | 536 | 12 | 1321 | 74 | 1 | FALSE | 3.23E-10 | 2.98E-07 | vesicle |  |
| GO:0044248 | 13453 | 316 | 536 | 38 | 5274 | 210 | 2 | FALSE | 3.60E-10 | 3.32E-07 | cellular catabolic process |  |
| GO:0006091 | 13453 | 96 | 536 | 20 | 5214 | 204 | 1 | FALSE | 4.41E-10 | 4.07E-07 | generation of precursor metabolites and energy |  |
| GO:0055086 | 13453 | 287 | 536 | 30 | 2376 | 77 | 2 | FALSE | 7.62E-10 | 7.02E-07 | nucleobase-containing small molecule metabolic process |  |
| GO:0007165 | 13453 | 268 | 536 | 23 | 1419 | 35 | 4 | FALSE | 9.27E-10 | 8.54E-07 | signal transduction |  |
| GO:0031410 | 13453 | 23 | 536 | 12 | 1477 | 91 | 3 | FALSE | 1.13E-09 | 1.04E-06 | cytoplasmic vesicle |  |
| GO:0012506 | 13453 | 23 | 536 | 12 | 2050 | 133 | 3 | FALSE | 2.50E-09 | 2.31E-06 | vesicle membrane |  |
| GO:0005737 | 13453 | 892 | 536 | 89 | 1864 | 121 | 1 | FALSE | 2.63E-09 | 2.42E-06 | cytoplasm |  |
| GO:0031090 | 13453 | 107 | 536 | 24 | 1741 | 104 | 3 | FALSE | 2.92E-09 | 2.69E-06 | organelle membrane |  |
| GO:0005198 | 13453 | 464 | 536 | 47 | 13352 | 535 | 1 | FALSE | 4.27E-09 | 3.94E-06 | structural molecule activity |  |
| GO:0046907 | 13453 | 133 | 536 | 30 | 704 | 59 | 2 | FALSE | 5.49E-09 | 5.05E-06 | intracellular transport |  |
| GO:0045184 | 13453 | 134 | 536 | 30 | 704 | 59 | 2 | FALSE | 6.73E-09 | 6.20E-06 | establishment of protein localization |  |
| GO:1901361 | 13453 | 168 | 536 | 24 | 2460 | 99 | 2 | FALSE | 1.53E-08 | 1.41E-05 | organic cyclic compound catabolic process |  |
| GO:0033036 | 13453 | 156 | 536 | 32 | 704 | 59 | 1 | FALSE | 1.64E-08 | 1.51E-05 | macromolecule localization |  |
| GO:0051641 | 13453 | 181 | 536 | 30 | 1402 | 84 | 2 | FALSE | 2.56E-08 | 2.36E-05 | cellular localization |  |
| GO:1901657 | 13453 | 188 | 536 | 26 | 6460 | 261 | 2 | FALSE | 2.67E-08 | 2.46E-05 | glycosyl compound metabolic process |  |
| GO:0044444 | 13453 | 694 | 536 | 74 | 1864 | 121 | 2 | FALSE | 2.93E-08 | 2.70E-05 | cytoplasmic part |  |
| GO:0044433 | 13453 | 23 | 536 | 12 | 1044 | 86 | 3 | FALSE | 2.96E-08 | 2.72E-05 | cytoplasmic vesicle part |  |
| GO:0019693 | 13453 | 188 | 536 | 26 | 2003 | 93 | 3 | FALSE | 1.10E-07 | 1.02E-04 | ribose phosphate metabolic process |  |
| GO:0012505 | 13453 | 62 | 536 | 17 | 2050 | 133 | 1 | FALSE | 1.41E-07 | 1.30E-04 | endomembrane system |  |
| GO:0043413 | 13453 | 32 | 536 | 9 | 1801 | 56 | 2 | FALSE | 2.27E-07 | 2.09E-04 | macromolecule glycosylation |  |
| GO:0006486 | 13453 | 32 | 536 | 9 | 1752 | 56 | 3 | FALSE | 2.87E-07 | 2.64E-04 | protein glycosylation |  |
| GO:1901265 | 13453 | 3395 | 536 | 142 | 6039 | 193 | 2 | FALSE | 3.09E-07 | 2.85E-04 | nucleoside phosphate binding |  |
| GO:0019637 | 13453 | 318 | 536 | 33 | 5967 | 240 | 2 | FALSE | 3.44E-07 | 3.17E-04 | organophosphate metabolic process |  |
| GO:0005794 | 13453 | 41 | 536 | 13 | 1269 | 81 | 2 | FALSE | 4.63E-07 | 4.26E-04 | Golgi apparatus |  |
| GO:0051179 | 13453 | 704 | 536 | 59 | 7776 | 343 | 1 | FALSE | 7.54E-07 | 6.95E-04 | localization |  |
| GO:0051234 | 13453 | 704 | 536 | 59 | 7776 | 343 | 2 | FALSE | 7.54E-07 | 6.95E-04 | establishment of localization |  |
| GO:0010646 | 13453 | 46 | 536 | 9 | 1297 | 33 | 2 | FALSE | 8.11E-07 | 7.47E-04 | regulation of cell communication |  |
| GO:0009101 | 13453 | 32 | 536 | 9 | 1860 | 67 | 3 | FALSE | 8.61E-07 | 7.93E-04 | glycoprotein biosynthetic process |  |
| GO:0048583 | 13453 | 46 | 536 | 9 | 1637 | 43 | 2 | FALSE | 1.35E-06 | 0.001240377 | regulation of response to stimulus |  |
| GO:0023051 | 13453 | 46 | 536 | 9 | 1346 | 37 | 2 | FALSE | 1.73E-06 | 0.001594895 | regulation of signaling |  |
| GO:0009100 | 13453 | 32 | 536 | 9 | 4771 | 181 | 3 | FALSE | 1.76E-06 | 0.001621106 | glycoprotein metabolic process |  |
| GO:0006753 | 13453 | 263 | 536 | 28 | 1959 | 85 | 3 | FALSE | 1.79E-06 | 0.0016526 | nucleoside phosphate metabolic process |  |
| GO:0006412 | 13453 | 529 | 536 | 37 | 3894 | 128 | 3 | FALSE | 3.03E-06 | 0.0027913 | translation |  |
| GO:0009894 | 13453 | 40 | 536 | 10 | 1589 | 69 | 2 | FALSE | 3.70E-06 | 0.003410381 | regulation of catabolic process |  |
| GO:1901137 | 13453 | 90 | 536 | 15 | 2437 | 105 | 2 | FALSE | 3.75E-06 | 0.003452045 | carbohydrate derivative biosynthetic process |  |
| GO:0015980 | 13453 | 10 | 536 | 6 | 834 | 48 | 2 | FALSE | 4.70E-06 | 0.004328222 | energy derivation by oxidation of organic compounds |  |
| GO:0030163 | 13453 | 136 | 536 | 19 | 2951 | 131 | 2 | FALSE | 5.08E-06 | 0.004675644 | protein catabolic process |  |
| GO:0071702 | 13453 | 205 | 536 | 33 | 704 | 59 | 1 | FALSE | 6.05E-06 | 0.005570802 | organic substance transport |  |
| GO:0044431 | 13453 | 41 | 536 | 13 | 1044 | 86 | 3 | FALSE | 8.64E-06 | 0.007958677 | Golgi apparatus part |  |
| GO:0051649 | 13453 | 179 | 536 | 30 | 704 | 59 | 2 | FALSE | 9.98E-06 | 0.009190069 | establishment of localization in cell |  |
| GO:0006099 | 13453 | 9 | 536 | 5 | 5818 | 239 | 2 | FALSE | 1.24E-05 | 0.011376636 | tricarboxylic acid cycle |  |
| GO:0001882 | 13453 | 2877 | 536 | 121 | 6050 | 194 | 4 | FALSE | 1.76E-05 | 0.016244292 | nucleoside binding |  |
| GO:0016192 | 13453 | 96 | 536 | 20 | 704 | 59 | 1 | FALSE | 2.50E-05 | 0.023021675 | vesicle-mediated transport |  |
| GO:0044267 | 13453 | 2538 | 536 | 109 | 4534 | 152 | 2 | FALSE | 3.61E-05 | 0.033289347 | cellular protein metabolic process |  |
| GO:0044265 | 13453 | 126 | 536 | 15 | 4398 | 159 | 3 | FALSE | 3.85E-05 | 0.035419411 | cellular macromolecule catabolic process |  |
| GO:0009057 | 13453 | 191 | 536 | 20 | 4854 | 190 | 2 | FALSE | 4.52E-05 | 0.041630915 | macromolecule catabolic process |  |

Supplementary Table S5. The list of differentially expressed genes among five tissues

(Please see the supplementary Excel file).

| Supplementary Table S6. The list of unique DGEs in the all sets of DGEs | | | | |
| --- | --- | --- | --- | --- |
| **Gene** | **Sample 1** | **Sample 2** | **Log_2_ (fold change)** | **q-value** |
| PH01001381G0520 | RT | SH | -5.0935 | 0.0423761 |
| PH01001103G0600 | RT | SH | -7.98016 | 0.0112803 |
| PH01001730G0080 | LF | SH | -4.87485 | 0.0487897 |
| PH01002410G0100 | RH | LF | 7.04939 | 0.0497007 |
| PH01000311G0660 | RT | PN | 5.58912 | 0.0457053 |
| PH01000019G2170 | RT | LF | -7.37626 | 0.0282213 |
| PH01000166G0130 | RT | LF | -4.86172 | 0.0280375 |
| PH01000908G0170 | RT | LF | -6.19277 | 0.0404617 |
| PH01000432G0670 | RT | LF | 5.57962 | 0.0369442 |
| PH01005948G0060 | RT | PN | 6.80106 | 0.0278349 |
| PH01001359G0510 | RT | LF | 4.614 | 0.0404292 |
| PH01000068G1440 | RT | LF | -7.0779 | 0.0331567 |
| PH01000191G0410 | RT | LF | -7.3742 | 0.0128125 |
| PH01000295G0040 | RT | LF | -5.96955 | 0.0447165 |
| PH01000346G0050 | RT | LF | -7.82846 | 0.0284755 |
| PH01000770G0470 | RT | LF | -5.53629 | 0.0280485 |
| PH01001342G0160 | RT | LF | -4.68723 | 0.0287096 |
| PH01001579G0330 | RT | LF | -6.96528 | 0.00332407 |
| PH01001844G0350 | RT | LF | -7.15725 | 0.0294218 |
| PH01002245G0190 | RT | LF | -7.30461 | 0.00930155 |
| PH01002424G0210 | PN | SH | 5.41581 | 0.0375722 |
| PH01000538G0650 | RH | LF | -5.77363 | 0.0332422 |
| PH01000646G0420 | RH | LF | -6.07556 | 0.0483755 |
| PH01000829G0650 | RH | LF | -7.69576 | 0.0336416 |
| PH01001477G0260 | RH | LF | -5.51306 | 0.0352584 |
| PH01001592G0130 | RH | LF | -6.59637 | 0.0380946 |
| PH01000674G0710 | RH | PN | 7.06028 | 0.0490186 |
| PH01001272G0030 | RH | LF | 5.8322 | 0.0339424 |
| PH01006983G0030 | RH | LF | -5.54944 | 0.00950638 |
| PH01000004G3640 | RT | LF | 7.03774 | 0.0331865 |
| PH01000005G1320 | RT | LF | 5.91335 | 0.0306698 |
| PH01000060G1700 | RT | LF | 6.75931 | 0.0154707 |
| PH01000167G0500 | RT | LF | 6.42061 | 0.0490663 |
| PH01000180G0230 | RT | LF | 6.98688 | 0.0147518 |
| PH01000210G0260 | RT | LF | 5.38113 | 0.00932503 |
| PH01000242G1010 | RT | LF | 7.41547 | 0.045681 |
| PH01000266G0910 | RT | LF | 7.36906 | 0.0468809 |
| PH01000298G0270 | RT | LF | 6.65085 | 0.0351307 |
| PH01000352G0320 | RT | LF | 7.96712 | 0.0230235 |
| PH01000366G0980 | RT | LF | 7.4207 | 0.0443355 |
| PH01000437G0390 | RT | LF | 5.80474 | 0.0482181 |
| PH01000467G0560 | RT | LF | 7.68134 | 0.0215845 |
| PH01000669G0620 | RT | LF | 7.82683 | 0.030629 |
| PH01000818G0620 | RT | LF | 9.16163 | 0.00130142 |
| PH01000850G0240 | RT | LF | 6.85111 | 0.0252498 |
| PH01001245G0190 | RT | LF | 5.02576 | 0.041758 |
| PH01001258G0290 | RT | LF | 6.77746 | 0.0469499 |
| PH01001350G0200 | RT | LF | 7.4163 | 0.0463405 |
| PH01001657G0190 | RT | LF | 6.17957 | 0.0484414 |
| PH01001777G0070 | RT | LF | 6.34489 | 0.0246077 |
| PH01001976G0230 | RT | LF | 6.2415 | 0.024112 |
| PH01002132G0200 | RT | LF | 8.33355 | 0.0137385 |
| PH01002295G0010 | RT | LF | 7.89062 | 0.0246726 |
| PH01003057G0090 | RT | LF | 6.72566 | 0.0499466 |
| PH01003344G0050 | RT | LF | 7.41262 | 0.0434834 |
| PH01004948G0090 | RT | LF | 6.77302 | 0.0454781 |
| PH01005718G0070 | RT | LF | 6.47262 | 0.0442313 |
| PH01006812G0030 | RT | LF | 6.58857 | 0.0271919 |
| PH01080680G0010 | RT | LF | 6.57029 | 0.0213756 |
| PH01081003G0010 | RT | LF | 7.50389 | 0.0449731 |
| PH01000059G1030 | RH | LF | 7.12475 | 0.0464277 |
| PH01000059G1270 | RH | LF | 5.50566 | 0.0434327 |
| PH01000133G0330 | RH | LF | 7.30962 | 0.0356937 |
| PH01000139G0020 | RH | LF | 6.67132 | 0.0385 |
| PH01000213G1410 | RH | LF | 7.33509 | 0.0347684 |
| PH01000254G0780 | RH | LF | 5.30571 | 0.0290495 |
| PH01000471G0220 | RH | LF | 7.20351 | 0.0406477 |
| PH01000500G0520 | RH | LF | 6.10248 | 0.0353831 |
| PH01002449G0230 | RH | LF | 4.53719 | 0.0464277 |
| PH01005750G0010 | RH | LF | 7.3271 | 0.0360863 |
| PH01000048G0900 | RT | PN | -6.76544 | 0.0443041 |
| PH01000019G0470 | RT | PN | 4.32825 | 0.0467274 |
| PH01000151G0410 | RT | PN | -7.19762 | 0.0242733 |
| PH01000046G0060 | RT | PN | 4.50511 | 0.0400818 |
| PH01000117G0310 | RT | PN | 8.1323 | 0.0280993 |
| PH01000156G0850 | RT | PN | 6.00573 | 0.0444821 |
| PH01000174G0590 | RT | PN | 4.40405 | 0.0437761 |
| PH01000353G0210 | RT | PN | 6.11794 | 0.0165402 |
| PH01001032G0370 | RT | PN | -9.20789 | 0.0110019 |
| PH01001051G0480 | RT | PN | -6.50659 | 0.0286964 |
| PH01001236G0310 | RT | PN | -7.70482 | 0.0113485 |
| PH01001046G0030 | RT | PN | 7.11941 | 0.0284134 |
| PH01001070G0190 | RT | PN | 7.38475 | 0.04664 |
| PH01001765G0020 | RT | PN | -8.04259 | 0.00678347 |
| PH01001751G0380 | RT | PN | 7.63676 | 0.0341032 |
| PH01002296G0200 | RT | PN | -5.16469 | 0.0180802 |
| PH01001859G0290 | RT | PN | 7.73558 | 0.0114346 |
| PH01001914G0150 | RT | PN | 6.0776 | 0.0132628 |
| PH01001963G0320 | RT | PN | 7.39546 | 0.0462599 |
| PH01002033G0280 | RT | PN | 6.67608 | 0.0331786 |
| PH01002396G0160 | RT | PN | 7.39753 | 0.0458811 |
| PH01005803G0010 | RT | PN | -5.30615 | 0.0444109 |
| PH01003175G0110 | RT | PN | 6.18369 | 0.0483491 |
| PH01004112G0160 | RT | PN | 6.97681 | 0.0350295 |
| PH01000061G0380 | RH | PN | -4.41164 | 0.0487191 |
| PH01000034G0340 | RH | PN | 5.2006 | 0.0340717 |
| PH01000095G1340 | RH | PN | 7.1767 | 0.0420111 |
| PH01000164G0470 | RH | PN | 5.5416 | 0.0454453 |
| PH01001133G0100 | RH | PN | 4.7773 | 0.0482353 |
| PH01001848G0230 | RH | PN | -7.19933 | 0.0241232 |
| PH01001737G0180 | RH | PN | 6.21106 | 0.0455974 |
| PH01002701G0030 | RH | PN | 5.8695 | 0.0437301 |
| PH01004855G0070 | RH | PN | 6.13032 | 0.0240944 |
| PH01006029G0090 | RT | SH | -6.31875 | 0.0484414 |
| PH01002285G0270 | RT | SH | -7.23358 | 0.0152915 |
| PH01000314G0710 | RT | RH | 5.73925 | 0.0263744 |
| PH01000570G0800 | RT | RH | 4.75468 | 0.0456155 |
| PH01000739G0580 | RT | RH | -6.39597 | 0.0348799 |
| PH01000894G0670 | RT | RH | 7.51602 | 0.040252 |
| PH01001072G0150 | RT | RH | -5.43599 | 0.0303068 |
| PH01001359G0550 | RT | RH | 5.20576 | 0.0485064 |
| PH01002810G0020 | RT | RH | 5.10815 | 0.0462826 |
| PH01003947G0110 | RT | RH | -6.68758 | 0.0227303 |
| PH01004475G0060 | RT | RH | -6.69929 | 0.0358361 |
| PH01004477G0070 | RT | RH | 5.61624 | 0.0147566 |
| PH01091676G0010 | RT | RH | -5.80685 | 0.0482036 |
| PH01000038G1480 | RT | SH | -8.12611 | 0.0252847 |
| PH01000093G1820 | RT | SH | -8.99394 | 0.0144058 |
| PH01000150G0460 | RT | SH | 5.0367 | 0.035604 |
| PH01000193G0900 | RT | SH | 7.6958 | 0.0314441 |
| PH01000354G0450 | RT | SH | -8.21506 | 0.0382164 |
| PH01000433G0370 | RT | SH | -6.95566 | 0.0093459 |
| PH01000490G1060 | RT | SH | 7.45259 | 0.0428304 |
| PH01000609G0140 | RT | SH | -6.52623 | 0.0109357 |
| PH01000615G0340 | RT | SH | -6.95832 | 0.041758 |
| PH01000688G0550 | RT | SH | -8.29419 | 0.0127442 |
| PH01000756G0110 | RT | SH | -7.51096 | 0.0376108 |
| PH01000851G0380 | RT | SH | -4.37755 | 0.0429619 |
| PH01000944G0560 | RT | SH | 5.66176 | 0.0349082 |
| PH01001090G0620 | RT | SH | -8.42019 | 0.0108053 |
| PH01001203G0150 | RT | SH | -6.18136 | 0.0417014 |
| PH01001206G0160 | RT | SH | 7.74448 | 0.0292169 |
| PH01001675G0220 | RT | SH | -7.60637 | 0.0306792 |
| PH01002320G0240 | RT | SH | -10.5601 | 0.0256583 |
| PH01002370G0230 | RT | SH | 7.55656 | 0.0147439 |
| PH01003694G0110 | RT | SH | -6.513 | 0.0356114 |
| PH01004026G0140 | RT | SH | -6.39736 | 0.0477179 |
| PH01000144G0480 | LF | SH | -8.73465 | 0.0198315 |
| PH01000144G1010 | LF | SH | -8.09032 | 0.0272717 |
| PH01000173G0700 | LF | SH | -8.52183 | 0.0279846 |
| PH01000268G0490 | LF | SH | -9.17409 | 0.0119609 |
| PH01000288G1230 | LF | SH | -8.21979 | 0.0220075 |
| PH01000297G0550 | LF | SH | -5.53053 | 0.0370994 |
| PH01000329G0930 | LF | SH | -6.98115 | 0.0402958 |
| PH01000552G0250 | LF | SH | -8.00945 | 0.0204786 |
| PH01000594G0200 | LF | SH | -8.01308 | 0.0476635 |
| PH01000707G0410 | LF | SH | -6.41488 | 0.0206512 |
| PH01000720G0530 | LF | SH | -6.5945 | 0.0493793 |
| PH01000764G0680 | LF | SH | -8.19102 | 0.0391107 |
| PH01000891G0520 | LF | SH | -7.8722 | 0.0171796 |
| PH01000999G0610 | LF | SH | -7.5409 | 0.0220084 |
| PH01001042G0500 | LF | SH | -4.84979 | 0.0366219 |
| PH01001430G0430 | LF | SH | -7.36023 | 0.0287368 |
| PH01001431G0100 | LF | SH | -8.97631 | 0.0145032 |
| PH01001494G0180 | LF | SH | -6.32754 | 0.0454309 |
| PH01002320G0300 | LF | SH | -7.00507 | 0.0314638 |
| PH01002614G0060 | LF | SH | -9.09473 | 0.00678785 |
| PH01002954G0010 | LF | SH | -9.10276 | 0.0125753 |
| PH01002987G0150 | LF | SH | -8.54844 | 0.0265599 |
| PH01003209G0010 | LF | SH | -7.7129 | 0.0414104 |
| PH01004392G0080 | LF | SH | -8.4649 | 0.0290888 |
| PH01006245G0040 | LF | SH | -9.83238 | 0.048069 |
| PH01009807G0010 | LF | SH | -8.07071 | 0.0123796 |
| PH01000060G0800 | PN | SH | -7.60051 | 0.048044 |
| PH01000065G1650 | PN | SH | -6.95447 | 0.0447592 |
| PH01000431G0620 | PN | SH | -5.1655 | 0.0492107 |
| PH01000950G0410 | PN | SH | -10.5805 | 0.0251846 |
| PH01000972G0120 | PN | SH | -4.44274 | 0.0448557 |
| PH01001216G0220 | PN | SH | -5.69004 | 0.03295 |
| PH01001287G0090 | PN | SH | -6.07637 | 0.0345234 |
| PH01001681G0100 | PN | SH | -4.68992 | 0.0448321 |
| PH01001934G0130 | PN | SH | -8.3984 | 0.011227 |
| PH01004727G0150 | PN | SH | 4.36052 | 0.044396 |

Supplementary Table S7: The list of transcription factor in bamboo

(Please see the supplementary Excel file).

| Supplementary Table S8. The number of GRAS in the different species. | | | | | |
| --- | --- | --- | --- | --- | --- |
| **Species** | **Number** | **Species** | **Number** | **Species** | **Number** |
| *Artemisia annua* | 25 | *Aquilegia coerulea* | 43 | *Arachis hypogaea* | 29 |
| *Azadirachta indica* | 68 | *Arabidopsis lyrata* | 37 | *Aegilops tauschii* | 47 |
| *Arabidopsis thaliana* | 37 | *Amborella trichopoda* | 43 | *Brachypodium distachyon* | 48 |
| *Brassica napus* | 37 | *Brassica oleracea* | 12 | *Brassica rapa* | 48 |
| *Capsicum annuum* | 36 | *Cicer arietinum* | 44 | *Cajanus cajan* | 60 |
| *Citrus clementina* | 48 | *Citrullus lanatus* | 37 | *Cucumis melo* | 37 |
| *Carica papaya* | 42 | *Capsella rubella* | 39 | *Cannabis sativa* | 54 |
| *Cucumis sativus* | 43 | *Citrus sinensis* | 49 | *Eucalyptus grandis* | 95 |
| *Fragaria vesca* | 51 | *Gossypium hirsutum* | 44 | *Glycine max* | 139 |
| *Gossypium raimondii* | 113 | *Helianthus annuus* | 9 | *Hordeum vulgare* | 74 |
| *Jatropha curcas* | 55 | *Lotus japonicus* | 50 | *Lactuca sativa* | 39 |
| *Linum usitatissimum* | 119 | *Musa acuminata* | 73 | *Malus domestica* | 127 |
| *Manihot esculenta* | 78 | *Mimulus guttatus* | 38 | *Medicago truncatula* | 61 |
| *Nelumbo nucifera* | 38 | *Nicotiana tabacum* | 29 | *Oryza barthii* | 47 |
| *Oryza brachyantha* | 54 | *Oryza glaberrima* | 57 | *Oryza punctata* | 55 |
| *Oryza sativa* subsp. *indica* | 63 | *Oryza sativa* subsp. *japonica* | 69 | *Picea abies* | 44 |
| *Pyrus bretschneideri* | 99 | *Phoenix dactylifera* | 42 | *Picea glauca* | 14 |
| *Phyllostachys edulis* | 59 | *Physcomitrella patens* subsp*. patens* | 49 | *Prunus mume* | 46 |
| *Picea sitchensis* | 5 | *Pinus taeda* | 9 | *Prunus persica* | 49 |
| *Ricinus communis* | 48 | *Raphanus sativus* | 17 | *Populus trichocarpa* | 151 |
| *Setaria italica* | 61 | *Solanum lycopersicum* | 54 | *Sorghum bicolor* | 86 |
| *Saccharum officinarum* | 42 | *Solanum tuberosum* | 71 | *Selaginella moellendorffii* | 54 |
| *Theobroma cacao* | 70 | *Thellungiella halophila* | 37 | *Triticum aestivum* | 56 |
| *Triticum urartu* | 25 | *Utricularia gibba* | 42 | *Thellungiella parvula* | 35 |
| *Vitis vinifera* | 43 | *Zea mays* | 104 | *Vigna unguiculata* | 9 |

Supplementary Table S9. Overview of the co-expression analysis

| Locus | Annotation | Pathways |
| --- | --- | --- |
| **PH01000073G1000** | GRAS family transcription factor domain containing protein | TF |
| **PH01004823G0070** | GRAS family transcription factor domain containing protein | TF |
| **PH01005217G0040** | GRAS family transcription factor domain containing protein | TF |
| PH01000002G3110 | auxin response factor | -- |
| PH01000004G3230 | transmembrane amino acid transporter protein | Plant hormone signal transduction |
| PH01000004G3230 | transmembrane amino acid transporter protein | Plant hormone signal transduction |
| PH01000005G0960 | zinc finger, C3HC4 type domain containing protein | -- |
| PH01000010G2350 | Citrate transporter protein | -- |
| PH01000019G0220 | heavy metal-associated domain containing protein | -- |
| PH01000022G1070 | Core histone H2A/H2B/H3/H4 domain containing protein | -- |
| PH01000026G0890 | aminotransferase, classes I and II, domain containing protein | -- |
| PH01000036G1560 | ABC transporter, ATP-binding protein | RNA degradation |
| PH01000038G0780 | ribosomal protein S2 | Ribosome |
| PH01000040G2010 | defender against cell death 1 | N-Glycan biosynthesis |
| PH01000042G0280 | ubiquitin carboxyl-terminal hydrolase, family 1 | -- |
| PH01000044G0140 | PDI | -- |
| PH01000045G1480 | acyl CoA binding protein | -- |
| PH01000046G0020 | drought induced 19 protein | -- |
| PH01000047G0400 | expressed_protein | -- |
| PH01000050G0740 | AN1-like zinc finger domain containing protein | -- |
| PH01000054G2280 | Core histone H2A/H2B/H3/H4 domain containing protein | -- |
| PH01000055G0990 | LSM domain containing protein | Spliceosome |
| PH01000078G1920 | pex14 | Peroxisome |
| PH01000081G1110 | YT521-B-like family domain containing protein | -- |
| PH01000083G0130 | NAC domain containing protein | TF |
| PH01000101G1130 | no apical meristem protein | TF |
| PH01000102G1710 | cystathionine gamma-synthase | Sulfur metabolism |
| PH01000110G0500 | helix-loop-helix DNA-binding domain containing protein | TF |
| PH01000117G0520 | no apical meristem protein | TF |
| PH01000118G0030 | uncharacterized protein At4g06744 precursor | -- |
| PH01000118G1190 | 60S ribosomal protein L23A | Ribosome |
| PH01000118G1190 | 60S ribosomal protein L23A | Ribosome |
| PH01000130G0820 | LTPL23 - Protease inhibitor/seed storage/LTP family protein precursor | -- |
| PH01000139G0840 | mannose-1-phosphate guanyltransferase | Fructose and mannose metabolism |
| PH01000140G0940 | signal peptide peptidase-like 2B | -- |
| PH01000182G1450 | dehydrogenase | Citrate cycle (TCA cycle) |
| PH01000190G1350 | VHS and GAT domain containing protein | -- |
| PH01000193G0970 | OsFBO13 - F-box and other domain containing protein | TF |
| PH01000210G1070 | AP2 domain containing protein | TF |
| PH01000240G0240 | splicing factor-related | -- |
| PH01000256G0260 | suppressor of G2 allele of SKP1 | -- |
| PH01000273G0040 | ZF-HD protein dimerisation region containing protein | TF |
| PH01000299G0430 | histidine-containing phosphotransfer protein | Plant hormone signal transduction |
| PH01000299G0660 | signal peptide peptidase domain containing protein | -- |
| PH01000300G0960 | expressed_protein | -- |
| PH01000309G1120 | histone H3 | -- |
| PH01000317G0460 | F-box/LRR-repeat protein 2 | -- |
| PH01000326G0530 | aspartic proteinase oryzasin-1 precursor | Lysosome |
| PH01000343G0770 | chaperone protein dnaJ | Protein processing in endoplasmic reticulum |
| PH01000366G0450 | amino acid transporter | -- |
| PH01000383G0320 | MYB family transcription factor | TF |
| PH01000386G0730 | RNA recognition motif | -- |
| PH01000406G0320 | expressed_protein | -- |
| PH01000410G0040 | OsClp8 - Putative Clp protease homologue | -- |
| PH01000417G0880 | 3-beta-hydroxysteroid-Delta-isomerase | -- |
| PH01001605G0350 | growth regulating factor protein | TF |

| Supplementary Table S10. RNA-Seq statistics using TopHat2 without the parameters of the expected inner distance and the standard deviation | | | | | | | | | | |
| --- | --- | --- | --- | --- | --- | --- | --- | --- | --- | --- |
|  | Root | | leaf | | rhizome | | panicle | | shoot | |
| Total reads | 84229772 | 100.00% | 75061534 | 100.00% | 73771970 | 100.00% | 83872774 | 100.00% | 285876190 | 100.00% |
| Total Basepairs | 8100321514 | 100.00% | 7940029694 | 100.00% | 7709128042 | 100.00% | 8065743156 | 100.00% | 27945422424 | 100.00% |
| Total Mapped reads | 77580713 | 92.11% | 62388922 | 83.12% | 61858411 | 83.85% | 74912152 | 89.32% | 265859924 | 93.00% |
| Fully match | 56630958 | 67.23% | 47302908 | 63.02% | 45619681 | 61.84% | 49592312 | 59.13% | 223965527 | 78.34% |
| Mismatch (<=2 bp) | 20949755 | 24.87% | 15086014 | 20.10% | 16238730 | 22.01% | 25319840 | 30.19% | 41894397 | 14.65% |
| Unique match | 26510939 | 31.47% | 26307063 | 35.05% | 18403655 | 24.95% | 23279409 | 27.76% | 101003614 | 35.33% |
| Multi-position match | 51069774 | 60.63% | 36081859 | 48.07% | 43454756 | 58.90% | 51632743 | 61.56% | 164856310 | 57.67% |
| Total unmatched reads | 6649059 | 7.89% | 12672612 | 16.88% | 11913559 | 16.15% | 8960622 | 10.68% | 20016266 | 7.00% |

Supplementary Table S11. The potential housekeeping genes in moso bamboo

(Please see the supplementary Excel file).

**Table S12. Transcriptional regulatory interactions predicted in moso bamboo**

| TF ID | | TF family | Target ID | | TF family | Activate/ |
| --- | --- | --- | --- | --- | --- | --- |
| Moso bamboo | Reciprocal best gene in Arabidopsis |  | Moso bamboo | Reciprocal best gene in Arabidopsis |  | Repress |
| PH01000383G0320 | AT1G01060 | MYB_related | PH01005551G0030 | AT5G15840 | CO-like | R |
| PH01000039G0180 | AT1G09530 | bHLH | PH01000383G0320 | AT1G01060 | MYB_related | A |
| PH01000958G0180 | AT1G09770 | MYB | PH01000392G1070 | AT1G62360 | TALE | A |
| PH01000958G0180 | AT1G09770 | MYB | PH01000057G1290 | AT2G17950 | WOX | A |
| PH01000011G0660 | AT1G19220 | ARF | PH01000788G0760 | AT3G58190 | LBD | A |
| PH01000057G1420 | AT1G19850 | ARF | PH01000057G1420 | AT1G19850 | ARF | A |
| PH01000057G1420 | AT1G19850 | ARF | PH01000135G0570 | AT3G20840 | AP2 | A |
| PH01000057G1420 | AT1G19850 | ARF | PH01001892G0010 | AT4G32880 | HD-ZIP | A |
| PH01000135G1070 | AT1G26870 | NAC | PH01000352G0610 | AT1G79580 | NAC | A |
| PH01000039G0640 | AT1G30490 | HD-ZIP | PH01000057G1290 | AT2G17950 | WOX | R |
| PH01000039G0640 | AT1G30490 | HD-ZIP | PH01002114G0160 | AT5G16560 | G2-like | R |
| PH01000003G1230 | AT1G32770 | NAC | PH01000820G0540 | AT1G28470 | NAC | A |
| PH01000003G1230 | AT1G32770 | NAC | PH01000043G1720 | AT1G62990 | TALE | A |
| PH01000003G1230 | AT1G32770 | NAC | PH01000569G0800 | AT1G66230 | MYB | A |
| PH01000003G1230 | AT1G32770 | NAC | PH01001133G0430 | AT1G73410 | MYB | A |
| PH01000003G1230 | AT1G32770 | NAC | PH01000060G0800 | AT4G12350 | MYB | A |
| PH01000003G1230 | AT1G32770 | NAC | PH01000008G3080 | AT4G22680 | MYB | A |
| PH01000003G1230 | AT1G32770 | NAC | PH01000298G0850 | AT4G28500 | NAC | A |
| PH01000003G1230 | AT1G32770 | NAC | PH01000006G2680 | AT5G12870 | MYB | A |
| PH01000048G1770 | AT1G62300 | WRKY | PH01000048G1770 | AT1G62300 | WRKY | R |
| PH01000392G1070 | AT1G62360 | TALE | PH01000071G1510 | AT1G65620 | LBD | R |
| PH01000392G1070 | AT1G62360 | TALE | PH01000053G1640 | AT1G76420 | NAC | R |
| PH01000392G1070 | AT1G62360 | TALE | PH01000369G0610 | AT2G37630 | MYB | R |
| PH01000071G1510 | AT1G65620 | LBD | PH01001344G0260 | AT1G32240 | G2-like | R |
| PH01000071G1510 | AT1G65620 | LBD | PH01000392G1070 | AT1G62360 | TALE | R |
| PH01000071G1510 | AT1G65620 | LBD | PH01000018G0940 | AT2G33860 | ARF | R |
| PH01000071G1510 | AT1G65620 | LBD | PH01001738G0010 | AT2G45190 | YABBY | R |
| PH01000071G1510 | AT1G65620 | LBD | PH01000001G1650 | AT4G00180 | YABBY | R |
| PH01000071G1510 | AT1G65620 | LBD | PH01002114G0160 | AT5G16560 | G2-like | R |
| PH01000071G1510 | AT1G65620 | LBD | PH01000357G1080 | AT5G63090 | LBD | A |
| PH01000029G0970 | AT1G68640 | bZIP | PH01000057G1290 | AT2G17950 | WOX | R |
| PH01000044G0380 | AT1G71930 | NAC | PH01000820G0540 | AT1G28470 | NAC | A |
| PH01000044G0380 | AT1G71930 | NAC | PH01000043G1720 | AT1G62990 | TALE | A |
| PH01000044G0380 | AT1G71930 | NAC | PH01000006G2680 | AT5G12870 | MYB | A |
| PH01000053G1640 | AT1G76420 | NAC | PH01000392G1070 | AT1G62360 | TALE | A |
| PH01000352G0610 | AT1G79580 | NAC | PH01000135G1070 | AT1G26870 | NAC | R |
| PH01000071G0980 | AT2G02450 | NAC | PH01005551G0030 | AT5G15840 | CO-like | R |
| PH01000006G0810 | AT2G03340 | WRKY | PH01000048G1770 | AT1G62300 | WRKY | A |
| PH01000057G1290 | AT2G17950 | WOX | PH01000392G1070 | AT1G62360 | TALE | A |
| PH01000057G1290 | AT2G17950 | WOX | PH01000007G1520 | AT4G37750 | AP2 | A |
| PH01000331G0630 | AT2G20180 | bHLH | PH01000437G0190 | AT2G36270 | bZIP | A |
| PH01000331G0630 | AT2G20180 | bHLH | PH01005548G0080 | AT5G08130 | bHLH | A |
| PH01001057G0130 | AT2G28610 | WOX | PH01000001G1650 | AT4G00180 | YABBY | R |
| PH01001751G0380 | AT2G33810 | SBP | PH01000306G0610 | AT5G60910 | MIKC | A |
| PH01000018G0940 | AT2G33860 | ARF | PH01001738G0010 | AT2G45190 | YABBY | A |
| PH01000018G0940 | AT2G33860 | ARF | PH01000740G0150 | AT4G36930 | bHLH | R |
| PH01000437G0190 | AT2G36270 | bZIP | PH01000437G0190 | AT2G36270 | bZIP | A |
| PH01000437G0190 | AT2G36270 | bZIP | PH01000050G1350 | AT5G56270 | WRKY | A |
| PH01000369G0610 | AT2G37630 | MYB | PH01001344G0260 | AT1G32240 | G2-like | R |
| PH01000369G0610 | AT2G37630 | MYB | PH01000018G0940 | AT2G33860 | ARF | R |
| PH01000369G0610 | AT2G37630 | MYB | PH01001738G0010 | AT2G45190 | YABBY | R |
| PH01000369G0610 | AT2G37630 | MYB | PH01000357G1080 | AT5G63090 | LBD | A |
| PH01000206G0250 | AT2G38470 | WRKY | PH01001112G0620 | AT3G15210 | ERF | R |
| PH01000206G0250 | AT2G38470 | WRKY | PH01000296G0390 | AT5G07100 | WRKY | A |
| PH01002127G0260 | AT2G42830 | MIKC | PH01001578G0450 | AT5G67110 | bHLH | A |
| PH01001738G0010 | AT2G45190 | YABBY | PH01002127G0260 | AT2G42830 | MIKC | A |
| PH01001738G0010 | AT2G45190 | YABBY | PH01000306G0610 | AT5G60910 | MIKC | A |
| PH01190367G0010 | AT3G03450 | GRAS | PH01000437G0190 | AT2G36270 | bZIP | A |
| PH01000769G0620 | AT3G13540 | MYB_related | PH01000014G2500 | AT1G79840 | HD-ZIP | A |
| PH01000530G0220 | AT3G20770 | EIL | PH01000343G0330 | AT5G47220 | ERF | A |
| PH01000002G3170 | AT3G26744 | bHLH | PH01000305G0990 | AT3G23250 | MYB | R |
| PH01000002G3170 | AT3G26744 | bHLH | PH01005069G0010 | AT5G53210 | bHLH | A |
| PH01005516G0020 | AT3G26790 | B3 | PH01000106G0210 | AT1G69180 | YABBY | A |
| PH01005516G0020 | AT3G26790 | B3 | PH01005516G0020 | AT3G26790 | B3 | A |
| PH01005516G0020 | AT3G26790 | B3 | PH01001622G0290 | AT5G67300 | MYB | A |
| PH01000001G1650 | AT4G00180 | YABBY | PH01000392G1070 | AT1G62360 | TALE | R |
| PH01000001G1650 | AT4G00180 | YABBY | PH01000071G1510 | AT1G65620 | LBD | R |
| PH01000001G1650 | AT4G00180 | YABBY | PH01000306G0610 | AT5G60910 | MIKC | A |
| PH01001363G0470 | AT4G00220 | LBD | PH01000392G1070 | AT1G62360 | TALE | A |
| PH01001363G0470 | AT4G00220 | LBD | PH01000369G0610 | AT2G37630 | MYB | R |
| PH01001232G0560 | AT4G01250 | WRKY | PH01001953G0270 | AT4G23550 | WRKY | A |
| PH01001953G0270 | AT4G23550 | WRKY | PH01001953G0270 | AT4G23550 | WRKY | A |
| PH01000224G1000 | AT4G31550 | WRKY | PH01000224G1000 | AT4G31550 | WRKY | A |
| PH01000007G1520 | AT4G37750 | AP2 | PH01000106G0210 | AT1G69180 | YABBY | R |
| PH01000007G1520 | AT4G37750 | AP2 | PH01001738G0010 | AT2G45190 | YABBY | A |
| PH01000007G1520 | AT4G37750 | AP2 | PH01000001G1650 | AT4G00180 | YABBY | A |
| PH01000007G1520 | AT4G37750 | AP2 | PH01000017G2520 | AT5G60690 | HD-ZIP | A |
| PH01000066G1200 | AT4G38620 | MYB | PH01000066G1200 | AT4G38620 | MYB | R |
| PH01000223G1160 | AT5G02030 | TALE | PH01002127G0260 | AT2G42830 | MIKC | R |
| PH01000223G1160 | AT5G02030 | TALE | PH01001578G0450 | AT5G67110 | bHLH | R |
| PH01000075G0960 | AT5G11260 | bZIP | PH01000437G0190 | AT2G36270 | bZIP | A |
| PH01000075G0960 | AT5G11260 | bZIP | PH01001892G0010 | AT4G32880 | HD-ZIP | A |
| PH01000006G2680 | AT5G12870 | MYB | PH01000043G1720 | AT1G62990 | TALE | A |
| PH01000006G2680 | AT5G12870 | MYB | PH01000008G3080 | AT4G22680 | MYB | A |
| PH01002114G0160 | AT5G16560 | G2-like | PH01000071G1510 | AT1G65620 | LBD | R |
| PH01000114G0050 | AT5G20730 | ARF | PH01000011G0660 | AT1G19220 | ARF | A |
| PH01000114G0050 | AT5G20730 | ARF | PH01000135G0570 | AT3G20840 | AP2 | A |
| PH01000114G0050 | AT5G20730 | ARF | PH01000788G0760 | AT3G58190 | LBD | A |
| PH01000014G1850 | AT5G40330 | MYB | PH01000014G2500 | AT1G79840 | HD-ZIP | A |
| PH01000014G1850 | AT5G40330 | MYB | PH01000014G1850 | AT5G40330 | MYB | A |
| PH01000083G0580 | AT5G41410 | TALE | PH01000057G1290 | AT2G17950 | WOX | R |
| PH01005069G0010 | AT5G53210 | bHLH | PH01000002G3170 | AT3G26744 | bHLH | A |
| PH01000050G1350 | AT5G56270 | WRKY | PH01000437G0190 | AT2G36270 | bZIP | A |
| PH01000306G0610 | AT5G60910 | MIKC | PH01002127G0260 | AT2G42830 | MIKC | R |
| PH01000306G0610 | AT5G60910 | MIKC | PH01001578G0450 | AT5G67110 | bHLH | R |
| PH01000002G3110 | AT5G62000 | ARF | PH01000007G1520 | AT4G37750 | AP2 | R |

| Supplementary Table S13. The output of Trinity based on 5 datasets of RNA-Seq in moso bamboo |
| --- |
| ################################ |
| ## Counts of transcripts, etc. |
| ################################ |
| Total trinity 'genes': 163904 |
| Total trinity transcripts: 300902 |
| Percent GC: 46.36 |
| ######################################## |
| Stats based on ALL transcript contigs: |
| ######################################## |
| Contig N10: 3611 |
| Contig N20: 2788 |
| Contig N30: 2271 |
| Contig N40: 1873 |
| Contig N50: 1541 |
|  |
| Median contig length: 573 |
| Average contig: 928.98 |
| Total assembled bases: 279531079 |
| ##################################################### |
| ## Stats based on ONLY LONGEST ISOFORM per 'GENE': |
| ##################################################### |
| Contig N10: 3592 |
| Contig N20: 2710 |
| Contig N30: 2091 |
| Contig N40: 1564 |
| Contig N50: 1128 |
|  |
| Median contig length: 348 |
| Average contig: 676.82 |
| Total assembled bases: 110934007 |

Supplementary Table S14. Primers of 26 GRAS genes from moso bamboo used in qRT-PCR

| Gene | Locus | Forward primer (5' →3') | Reverse primer (5' → 3') |
| --- | --- | --- | --- |
| *PeGRAS-10* | PH01001692G0030 | ATGAGGGCGGCGCCCTTC | CCTTGTCCTCCGGCCAAAG |
| *PeGRAS-11* | PH01002325G0040 | ATGTCGTTGGTGCAACTGAAGG | CCTCCTCCCTTTCGCCTG |
| *PeGRAS-12* | PH01000860G0710 | ACATCCTTGCCGCACGAATTTG | GAGCGGAGGAAGGGCTTC |
| *PeGRAS-17* | PH01002322G0240 | ATGCATCACTGCCTCTACGACG | GTAGTGCAGCGCCTCCAC |
| *PeGRAS-2* | PH01005217G0040 | GAAGTCCAGTACCACCACGAAC | TAGCTGCAGAGGCGGTGG |
| *PeGRAS-20* | PH01000708G0250 | CTTTGACATCCTCCCCTGCG | CAGGTGGTGCTGCTCCTG |
| *PeGRAS-24* | PH01000712G0640 | AGGGCACGGAGGATCAATTGG | GCTATAGAGGTGAGCCTGGTG |
| *PeGRAS-27* | PH01000538G0420 | ATGTGGATGCTCAACGAGCTGG | AAGGACGACCAGGGGCTG |
| *PeGRAS-28* | PH01000073G0280 | AGCAGCAGCAGCAATCGGC | CCGTGCGAGTGGAAGTGC |
| *PeGRAS-29* | PH01000601G0200 | GAAGTTAGAGCACAAATCGCAAGG | CCGACGTCGACGAAGGCG |
| *PeGRAS-32* | PH01004866G0030 | ATGTCAGCTAAGGCTTCAAACC | CCTTCCATTTTCCCCTAAAGGC |
| *PeGRAS-34* | PH01001245G0190 | ATGGCTGATACTCCAACTTCCC | GAGGGAGAATCATGCCTCATAC |
| *PeGRAS-36* | PH01002417G0180 | AGCAAACTCTCCCTGGCCAG | TGGAGTGTTGCTAAGAGCAGAC |
| *PeGRAS-37* | PH01000382G0190 | GTCTTTTGTTAGGCAAGCAGATCC | GGACTGTCCAAAGCCCTCAG |
| *PeGRAS-39* | PH01000111G0510 | GGTGATGCTAAAACTTAATATGGG | GAACAACTCTGGACCTGCTAAG |
| *PeGRAS-42* | PH01000000G1660 | ACGTCTACGGGGTCGAATCG | CGAGGCTCCTCTTGAGCAC |
| *PeGRAS-43* | PH01000896G0440 | ATGGTGGCCGTTCTGTCCTC | CGGTGACGGCTATGTTAGCG |
| *PeGRAS-44* | PH01000054G0400 | ACGACTCGGAGGCGTTCTC | CGTCGATGTCCTCAGCCATG |
| *PeGRAS-45* | PH01000374G0830 | ATGGCCGAGGACATCGACG | GGAGTTGGTGACGCTACTGC |
| *PeGRAS-47* | PH01001761G0380 | TGGGTCTGGATAATACTCTTGGC | CCGGCTAATATAGTTGAGTGACTC |
| *PeGRAS-49* | PH01005526G0030 | ATGGAGGAGAACATCGACGACAAG | GGTTGTTGTTGCTGTCGGAGG |
| *PeGRAS-50* | PH01003146G0100 | ATGGCCACCACGCCGGAG | GTGTTGGTGGCAGGTCGAG |
| *PeGRAS-51* | PH01004053G0150 | ATGGGGAATGAGACCGGAAAGAAG | GGCGCAATGGACCAGCAG |
| *PeGRAS-59* | PH01004053G0220 | ATGGCTCCCTCTGCCGTC | CCTCCATGAGAATGCGCGAG |
| *PeGRAS-6* | PH01000397G0230 | ATGCCCTTAGCTGGTAGCGC | GACGTGGGCTCCCAGAAG |
| *PeGRAS-7* | PH01000287G0830 | ATGCAAGAGGGGAAGGCCG | GGGTCCCAGAAGCCGAAG |
